# Phase I Study of Intravitreal Injection of Autologous CD34+ Stem Cells from Bone Marrow in Eyes with Vision Loss from Retinitis Pigmentosa

**DOI:** 10.1016/j.xops.2024.100589

**Published:** 2024-07-31

**Authors:** Susanna S. Park, Gerhard Bauer, Brian Fury, Mehrdad Abedi, Nicholas Perotti, Dane Colead-Bergum, Jan A. Nolta

**Affiliations:** 1 Department of Ophthalmology & Vision Science, University of California Davis Eye Center, Sacramento, California; 2 Stem Cell Program, Institute for Regenerative Cures, University of California Davis Health, Sacramento, California; 3 Division of Hematology Oncology and Malignant Hematology/Cellular Therapy and Transplantation, Department of Internal Medicine, University of California Davis Health, Sacramento, California

**Keywords:** Retinal degeneration, Retinitis pigmentosa, Intravitreal stem cell therapy, Bone marrow stem cells, CD34+ cells

## Abstract

**Purpose:**

To evaluate the feasibility and safety of intravitreal injection of autologous CD34+ stem cells from bone marrow (BMSCs) in eyes with vision loss from retinitis pigmentosa (RP).

**Design:**

Phase I prospective, open-label, single-center study.

**Participants:**

Seven eyes (7 patients) with RP with best-corrected visual acuity (BCVA) of 20/60 to 20/400 or visual field constriction to within 10°.

**Methods:**

A comprehensive examination with ETDRS BCVA, macular OCT, perimetry, and fluorescein angiography was performed at baseline, 1 to 3 months, and 6 months after study treatment. Bone marrow aspiration, isolation of CD34+ BMSCs under good manufacturing practice conditions, and intravitreal cell injection were performed on the same day. The CD34+ cells were isolated from bone marrow using a Ficoll gradient and the Miltenyi CliniMACS system. Isolated CD34+ cells were released for clinical use if viability, sterility, and purity met the release criteria accepted by the United States Food and Drug Administration for this clinical study.

**Main Outcome Measures:**

Number of CD34+ cells isolated for injection and adverse events associated with study treatment during follow-up. Secondary outcome measures are changes in BCVA and perimetry.

**Results:**

All isolated CD34+ cells passed the release criteria. A mean of 3.26 ± 0.66 million viable CD34+ cells (range 1.6 to 7.05 million) were injected intravitreally per eye. No adverse event was noted during the study follow-up except for 1 participant who was noted with transient cells in the anterior chamber with mild elevation in intraocular pressure at 18 hours after study injection which normalized by 24 hours. Best-corrected visual acuity remained within 2 lines of baseline or improved in all participants at 6 months follow-up. Perimetry was stable or improved in all eyes during study follow-up except 1 eye with transient improvement at 1 month and worsening of both eyes at 6 months.

**Conclusions:**

Intravitreal injection of autologous CD34+ BMSCs is feasible and appears to be well tolerated in eyes with vision loss from RP. A larger randomized prospective study would be needed to evaluate further the safety and potential efficacy of this cell therapy for vision loss associated with RP.

**Financial Disclosure(s):**

Proprietary or commercial disclosure may be found in the Footnotes and Disclosures at the end of this article.

Retinitis pigmentosa (RP) represents a group of hereditary retinal disorders associated with diffuse photoreceptor degeneration and vision loss in both eyes. It affects about 1:4000 individuals worldwide.^1^ Patients present initially with loss of night and peripheral vision. Total blindness can result as the condition advances.

Currently, there are limited treatment options for RP. Despite published research supporting the use of nutritional supplementation, the effect of such nutritional supplementation on progression of RP is modest at best.^2^ In fact, recent reanalysis of 30-year-old research data showed no benefit of vitamin A palmitate supplementation, a nutritional supplement initially noted to be beneficial in slowing down progression of RP.^3^ Argus II retinal prosthesis was approved by the United States Food and Drug Administration (FDA) for advanced RP in 2013 for visual rehabilitation, but the device has been discontinued recently.^4^ Voretigene neparvovec was approved in 2017 as the first gene therapy for RP, but this adeno-associated viral vector-based gene therapy is a treatment option only for individuals with biallelic *RPE65*-mutated retinal dystrophy.^5^^,^^6^ These individuals make up <1% of patients with RP.^7^

Thus, a great unmet need remains to develop new therapies for RP. Because there are >100 different genes that have been identified to be associated with RP,^7^ a gene-agnostic approach would be particularly appealing. Stem cell therapy is such an approach. Stem and progenitor cells (of various origin, autologous, and allogeneic) can limit or reverse retinal degeneration by replacing degenerating retinal cells or via paracrine trophic effects.^8^ Currently, there are ≥2 different phase I/II clinical trials exploring subretinal transplantation of fetal neural progenitor cells or retinal progenitor cells for tissue replacement in eyes with RP.^9^ Intravitreal injection of fetal retinal progenitor cells is being explored in a phase II clinical trial for possible paracrine trophic effects in eyes with RP.^8^^,^^9^ These clinical trials use cultured allogeneic stem cells which can be expanded. However, rejection and abnormal cellular proliferation of these allogeneic cultured cells are potential safety concerns.^8^

Autologous stem cell therapy avoids rejection issues. Autologous stem cell therapy is possible using CD34+ stem cells. CD34+ stem cells are repair cells found mainly in bone marrow that get mobilized into the circulation in response to tissue injury; they home into damaged tissue and promote tissue repair via paracrine effect.^8^^,^^10^ Intracoronary infusion of autologous CD34+ stem cells from bone marrow has been explored in a phase II clinical trial showing safety and efficacy.^11^ By harvesting these repair cells directly from bone marrow and injecting the cell intravitreally, we aim to maximize the repair potential of these cells on degenerating or ischemic retina. Our group has explored intravitreal injection of human CD34+ stem cells from bone marrow (BMSCs) in immunocompromised rodent models of hereditary retinal degeneration and retinal vasculopathy.^12^, ^13^, ^14^ These studies demonstrated that intravitreal injection of human CD34+ BMSCs results in rapid retinal homing of the cells with protective effects on the retina or retinal vessels, depending on the underlying retinal pathology. In addition, no long-term ocular or systemic safety concerns were noted.^15^ We obtained an investigational new drug clearance from the FDA to explore intravitreal injection of autologous CD34+ BMSCs for retinopathy. A pilot phase I clinical trial has been conducted showing that a single intravitreal injection of autologous CD34+ BMSCs is feasible and well tolerated in eyes with vision loss from various retinal pathologies, including a patient with RP.^16^ Some efficacy signals were noted in visual function. In this follow-up study, we present the findings of the phase I clinical trial conducted to test the hypothesis that intravitreal injection of autologous CD34+ BMSCs is feasible and well tolerated in eyes with vision loss from RP.

## Methods

This phase I prospective, open-label, single-arm, single-center clinical trial was conducted at the University of California Davis Eye Center between January, 2014 and October, 2023. It was conducted under an investigational new drug cleared from the FDA (investigational new drug # 13307) and according to a clinical protocol approved by the FDA and the University of California Davis Office of Human Research (institutional review board) and Stem Cell Oversight Committee. It was conducted in adherence to the tenets of the Declaration of Helsinki. The study was listed in www.clinicaltrials.gov before enrollment (NCT01736059; NCT04925687). There was no randomization of this single-arm study.

The study enrolled 7 consecutive patients with RP who met study inclusion and exclusion criteria, agreed to participate in the study, and signed the informed consent form. The timing of the study enrollment was based on availability of research funding and identified study participants. There were some delays in enrollment during the coronavirus disease 2019 pandemic that limited participation and travel of study participants. There was no sample size calculation performed for this phase I study evaluating safety and feasibility. The sample size was based on available funding to support the study.

The study participants included patients diagnosed with RP in both eyes based on clinical examination and flat signals on electroretinography (ERG). In addition, syphilis serologies and fasting serum retinol levels were obtained to rule out treatable causes of retinal degeneration. All participants experienced progressive decrease in peripheral and night vision in both eyes for ≥6 months before study enrollment. Genetic testing was not required but encouraged during the course of the study. For study inclusion, patients needed to be ≥18 years of age, able to sign the study informed consent form, perform diagnosed tests outlined in the study protocol, and maintain study follow-up of 6 months. The study eye was the eye with worse best-corrected visual acuity (BCVA). If BCVA was equal in both eyes, the eye with greater visual field loss on perimetry was selected for study enrollment. The study enrollment vision criteria for the study eye initially were ETDRS BCVA of 20/100 to 20/400 or visual field constriction to 10° 360 or worse if BCVA was better than 20/100. Enrollment vision criteria were modified to BCVA 20/60 to 20/400 or visual field constriction to 10° or worse if BCVA was better than 20/60 during the course of the study to increase study enrollment. Enrollment exclusion criteria included concurrent retinal or optic nerve condition contributing to vision loss or concurrent systemic condition that would affect the components of bone marrow, including hematologic disorder, coagulopathy, active infection and concurrent immunosuppression. Eyes with nonangiographic cystoid macular edema due to RP were included in the study. We also included eyes with open-angle glaucoma or ocular hypertension if well controlled with 1 medication.

After obtaining informed consent, each participant had a comprehensive eye examination with ETDRS BCVA, full-field ERG, fundus photography, fluorescein angiography, macular OCT, and perimetry. For women of child bearing age, a urine pregnancy test was performed at baseline and study exit. Eye examination with ETDRS BCVA was repeated 1 day, 1 week, 1 month, 3 months, and 6 months after study treatment. Macular OCT was repeated at 1 month, 3 months, and 6 months after study treatment. Central macular thickness represents the thickness of zone 1 of the ETDRS macular thickness map on Cirrus OCT. The integrity of the macular photoreceptor layer was measured manually looking at the horizontal OCT line scan centered at the fovea and measuring the length of the intact inner segment–outer segment junction relative to the length of the entire OCT image using Harmony Medical software. Electroretinography and perimetry were repeated at 1 to 3 months and 6 months after study treatment. Fundus photography and fluorescein angiography were repeated at study exit at 6 months. During the course of the study, the protocol was modified to include the National Eye Institute Visual Function Questionnaire at baseline and at 6 months starting with participant #4. This change was based on volunteered information of subjective improvement in activities of daily living reported among the first 3 study participants.

**Figure 1:**
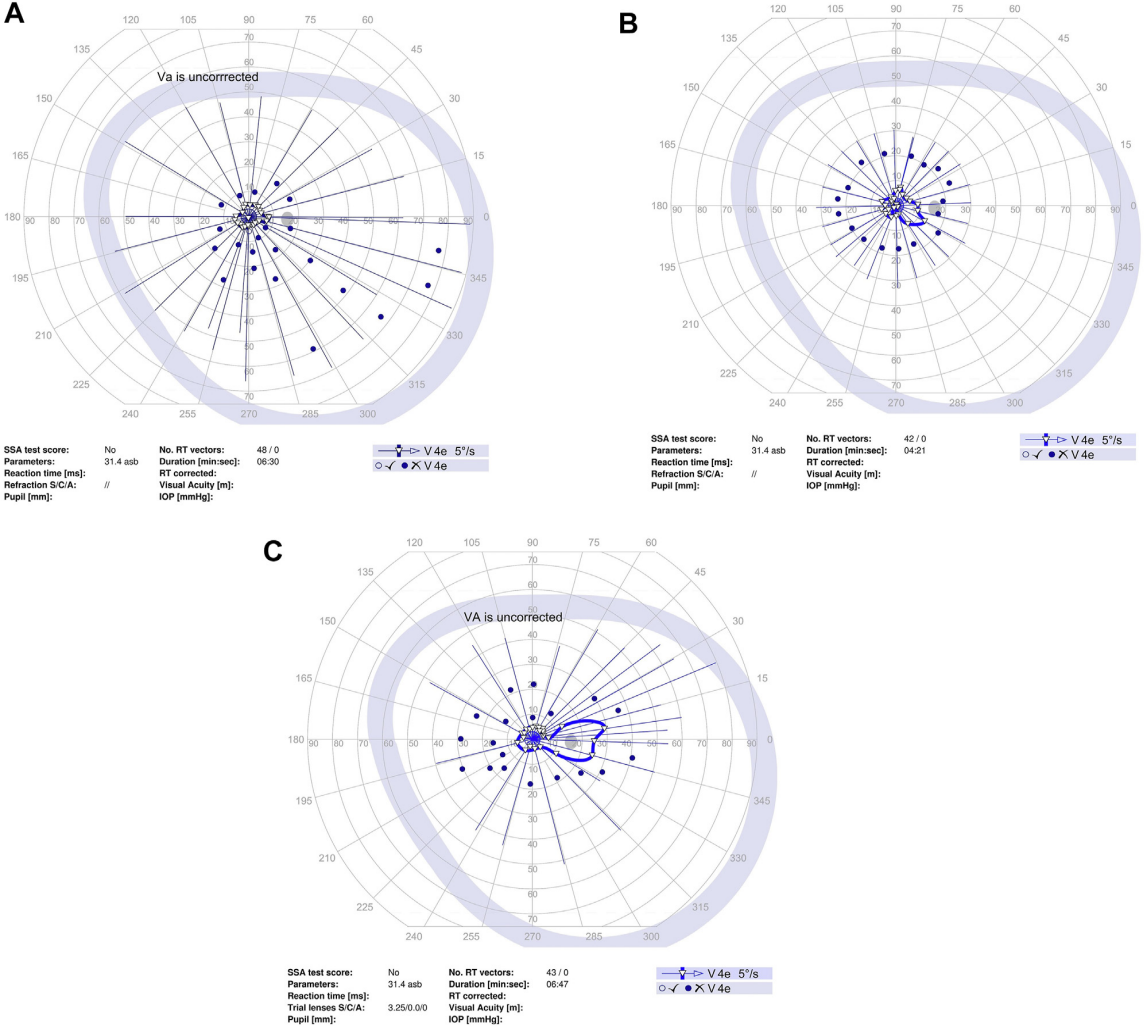

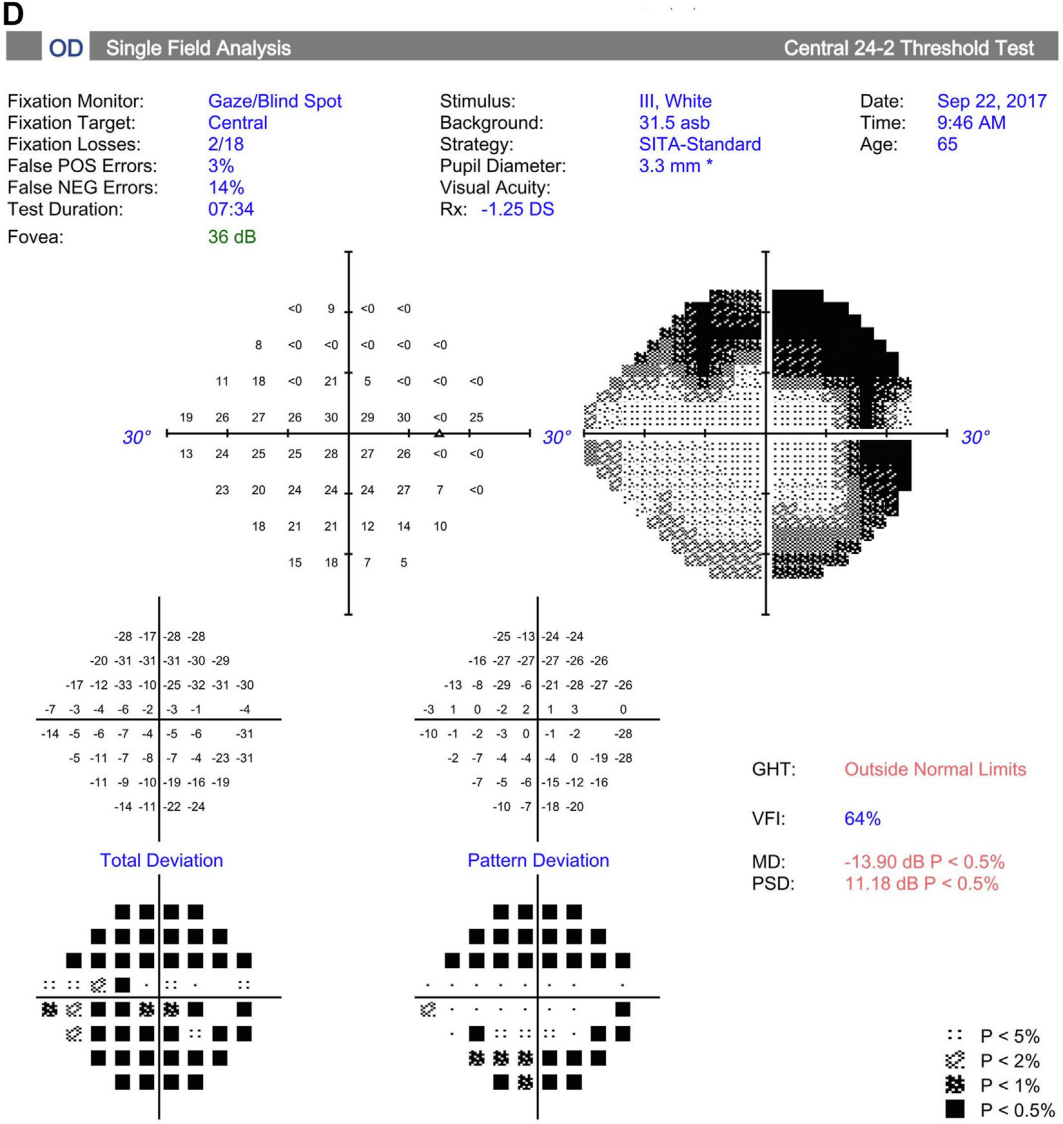

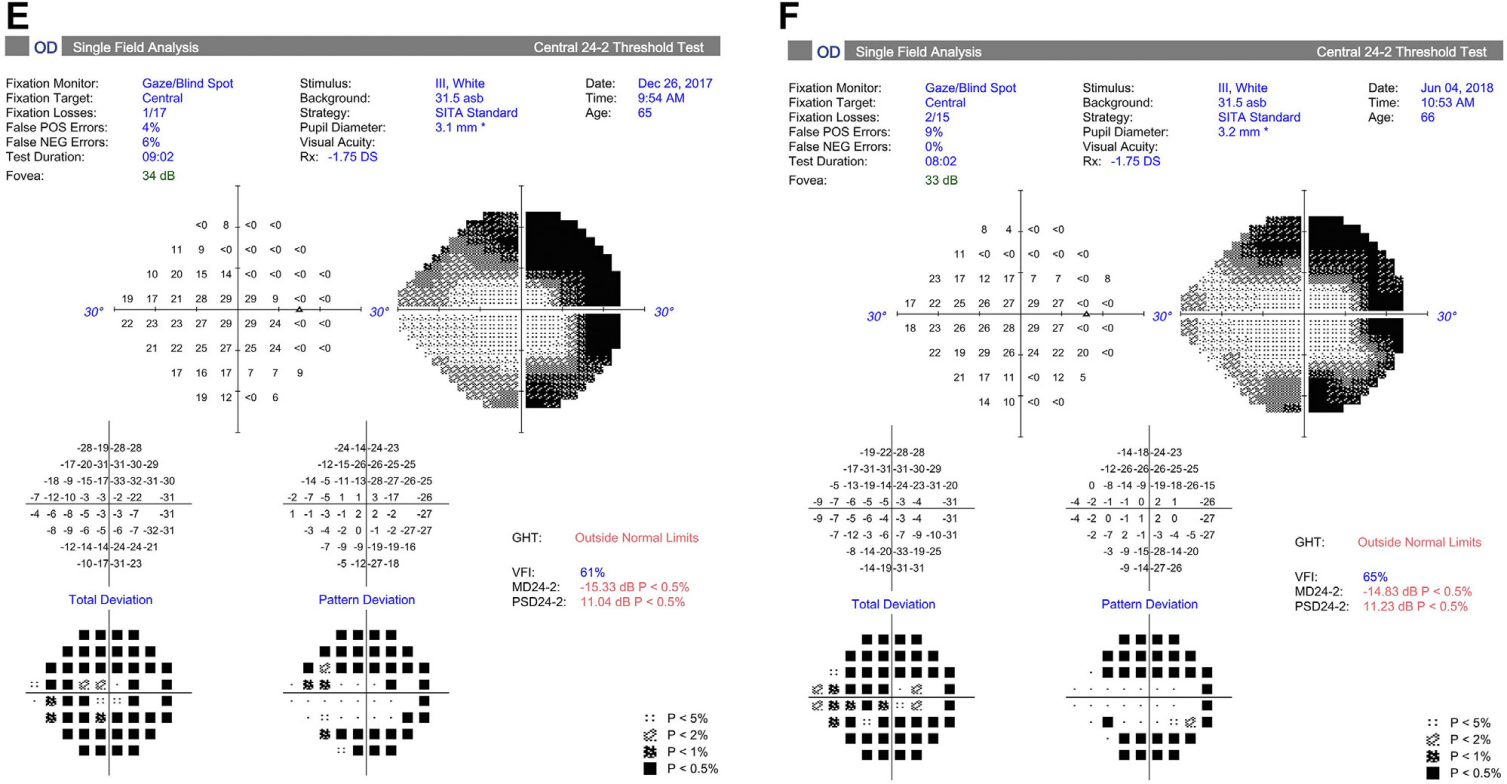

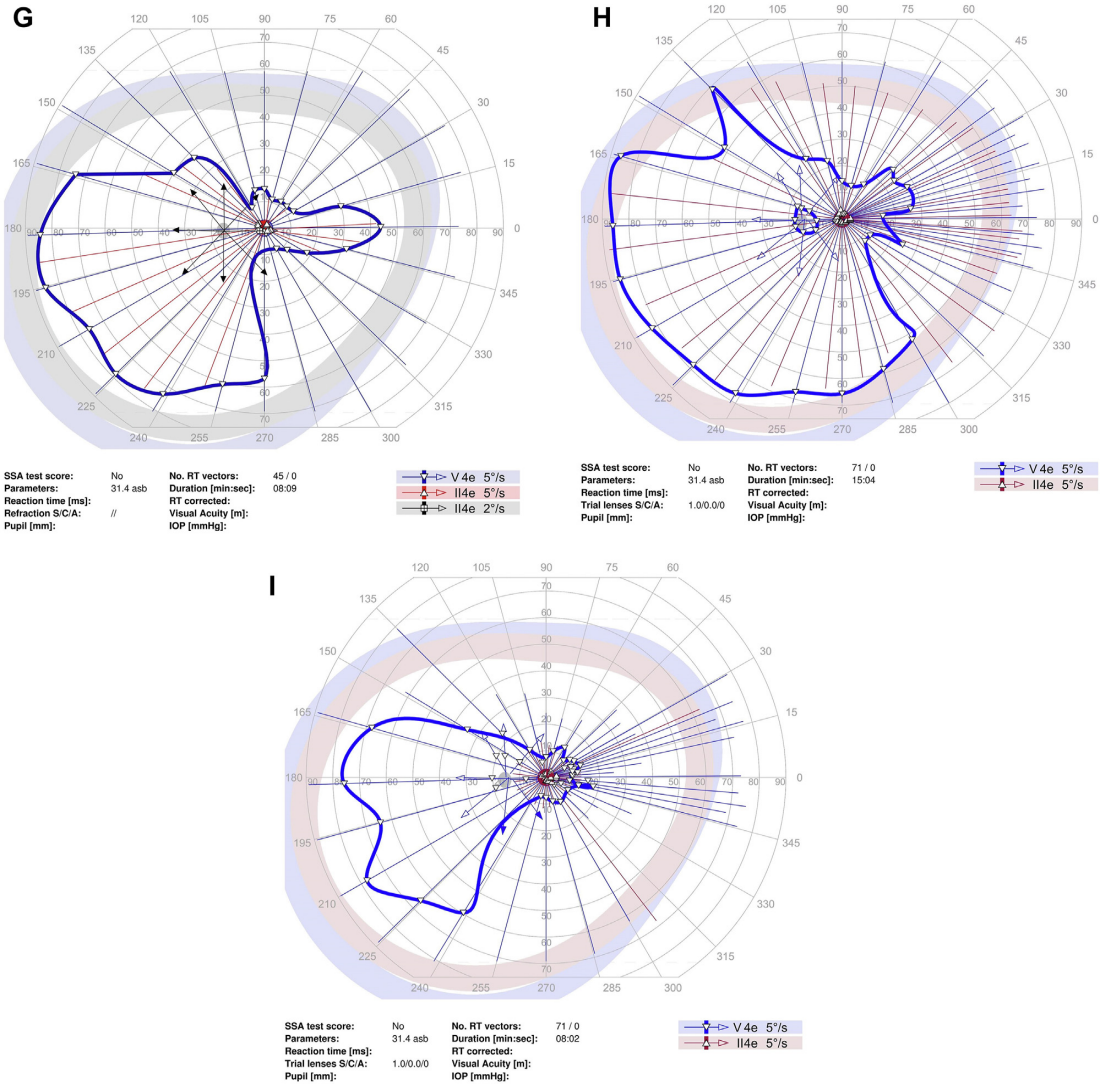

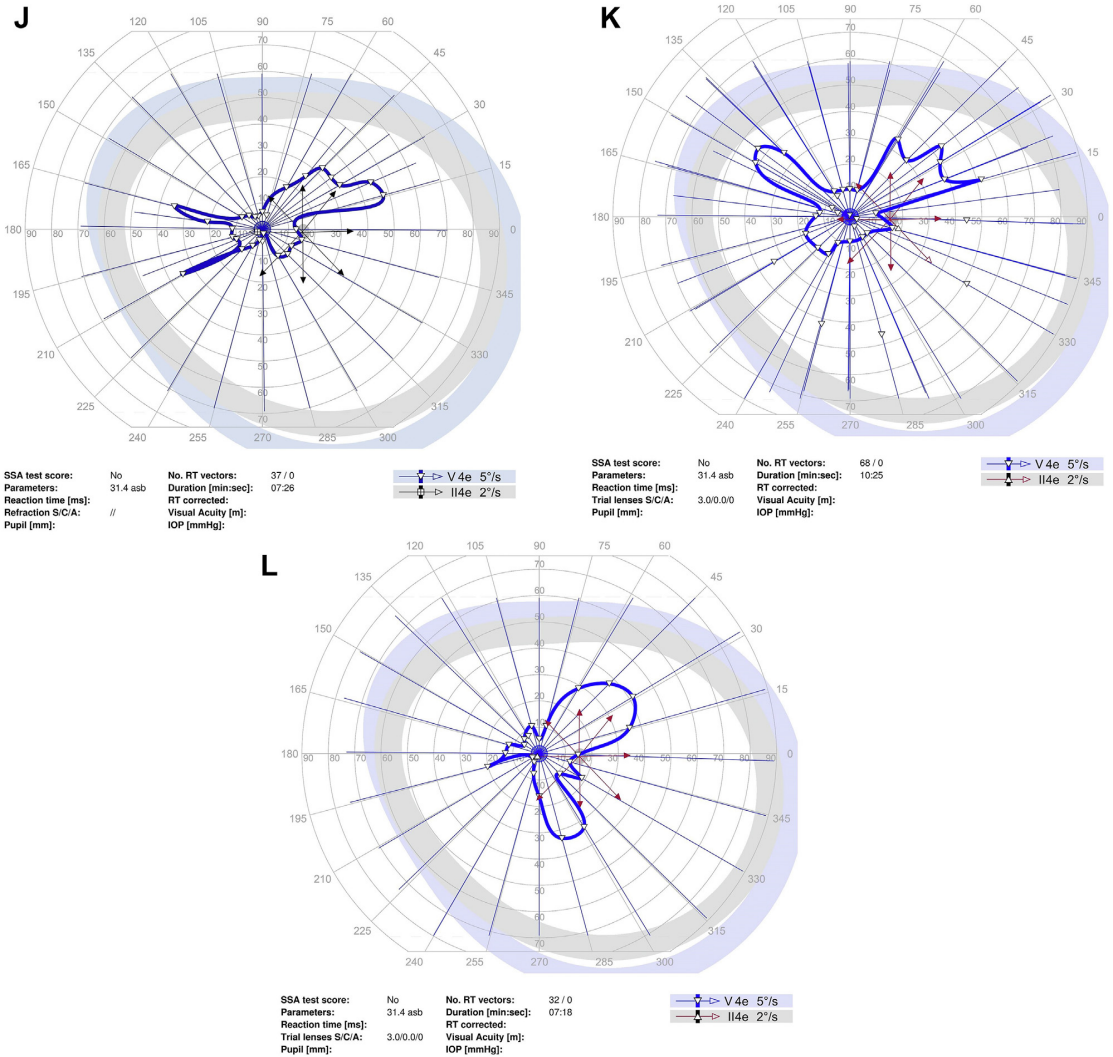

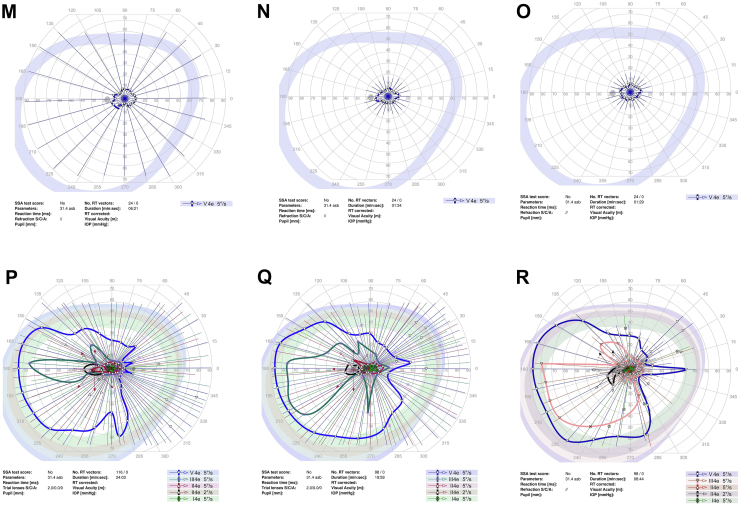
Perimetry of study eye at baseline and study follow-up for study participants. **A–C,** Goldmann perimetry for participant #2 at baseline (**A**), 3 months (**B**), and 6 months (**C**). **D–F,** Humphrey perimetry 24-2 for participant #3 at baseline (**D**), 1 month (**E**), and 6 months (**F**). **G–I,** Goldmann perimetry for participant #4 at baseline (**G**), 1.5 months (**H**), and 6 months (**I**). **J–L,** Goldmann perimetry for participant #5 at baseline (**J**), 3 month (**K**), and 6 months (**L**). **M–O,** Goldmann perimetry for participant #6 at baseline (**M**), 1 month (**N**), and 6 months (**O**). **P–R,** Goldmann perimetry for participant #7 at baseline (**P**), 1 month (**Q**), and 6 months (**R**). dB = decibels; GHT = glaucoma hemifield test; IOP = intraocular pressure; MD = mean deviation; NEG = negative; OD = right eye; POS = positive; PSD = pattern standard deviation; RT = reaction time; SSA = Social Security Administration; Trial lenses S/C/A = sphere/cylinder/axis; VA = visual acuity; VFI = visual field index.

Goldmann perimetry was performed at baseline and study follow-up for all study participants except for 1 participant who had Humphrey 24-2 perimetry (Fig 1). To quantitate the sensitivity on Goldmann perimetry, the % area of sensitivity for tested isopters, i.e., V4e and III4e (when available), was obtained by manually outlining the area of sensitivity and dividing the area by the total area of testing, i.e., the outer border of the purple zone outlined in the perimetry. This was done by viewing the uploaded perimetry images in the electronic medical record using Harmony Medical software which automatically quantitates the area of interest outlined manually.

Study treatment was a 1-day procedure consisting of bone marrow aspiration by the study hematologist (M.A.) under local anesthesia as an outpatient, isolation of the CD34+ BMSCs performed in the certified good manufacturing practice laboratory at the University of California Davis Institute for Regenerative Cures, and intravitreal injection of isolated CD34+ BMSC in the study eye by the study retinal specialist (S.S.P.) as outlined previously.^14^ For each study participant, 50 to 100 ml of bone marrow was aspirated from the iliac crest under local anesthesia and promptly transported at 4 ° C to the good manufacturing practice laboratory for CD34+ cell isolation. CD34+ cells were isolated from the bone marrow aspirate using a Ficoll density gradient to obtain the mononuclear cell fraction, followed by positive selection of CD34+ cells from the mononuclear cell fraction using the Miltenyi CliniMACS system. The isolated cells were washed with sterile balanced salt solution and tested for immediate release by applying a stat gram stain and stat endotoxin assay. The isolated CD34+ cells were released for intravitreal injection if they passed the release criteria accepted by FDA; i.e., the number of cells available for intravitreal injection was >5000, an absence of visible microorganisms was confirmed by a negative gram stain, a stat endotoxin assay confirmed as acceptable endotoxin result, and the viability of the isolated cells was >70% by trypan blue staining. A 14-day sterility test was set up at the same time, and a positive culture action plan was in place, in case the 14-day sterility assay showed growth at the specific read time points. The released, final cell product was suspended in 0.1 ml of sterile saline for eye injection and transported at 4 ° C to the eye clinic in a tuberculin syringe. Intravitreal injection of the isolated CD34+ BMSCs was performed in the study eye under local anesthesia using a 30-gauge needle and within 2 hours of cell release.

A fraction of the final cell product was saved for further analysis after release of the isolated cells for intravitreal injection. The postrelease analysis of the final cell product included flow cytometry to determine % CD34+ cells and % CD3+ cells.

The primary outcome measure for safety is any adverse event associated with study treatment or bone marrow aspiration during the 6 months study follow-up. The primary outcome measure for feasibility is number and purity of CD34+ cells isolated from bone marrow aspirate for intravitreal injection. The secondary outcome measures include changes in BCVA and perimetry at 1 to 3 months and 6 months study follow-up. A secondary outcome measure of change in vision questionnaire at 6 months when compared with baseline was also added during the course of the study. The stop criteria for the study enrollment included any serious adverse event directly attributed to study treatment including any eye with development of any intraocular tumor during the course of the study, any eye with severe intraocular inflammation or endophthalmitis associated with >3 lines of drop in BCVA and positive 14-day culture despite passing release criteria or ≥2 eyes with severe intraocular inflammation or endophthalmitis with >3 lines of drop in BCVA and negative 14-day culture, drop in BCVA of >3 lines not attributed to normal progression of RP in ≥2 participants during the study follow-up, new retinal vascular occlusion in ≥2 participants during the study follow-up, new ocular neovascularization during the study follow-up noted in ≥2 participants and not attributed to normal progression of RP, sustained elevation of intraocular pressure >30 mmHg not controlled with medication or paracentesis and directly attributed to study therapy, and any adverse event resulting in enucleation or loss of eye directly attributed to the study therapy.

## Results

Seven eyes of 7 patients with RP were enrolled in the study, including 1 participant who had been reported previously in the preliminary phase I study data (participant #1).^16^ The baseline demographic and clinical characteristics of the study participants are outlined in Table 1. None of the participants had a family history of RP or any other type of inherited retinal dystrophy. The diagnosis of RP was confirmed at baseline based on flat ERG in both eyes, fundus finding of diffuse peripheral retinal degeneration including bone spicules in both eyes, history of progressive loss of night and peripheral vision in both eyes, and negative testing for malabsorption (based on normal fasting serum retinol) and infection (negative syphilis serologies). Figure S1 (available at www.ophthalmologyscience.org) shows the fundus photography of all study eyes at baseline and 6 months follow-up. Figure S2 (available at www.ophthalmologyscience.org) shows macular OCT of the study eyes at baseline and 6 months follow-up.

**Table 1:** Baseline Demographics and Clinical Characteristics of Study Participants

Participant	Age/Sex/Study Eye	Race	POH
1	36/male/OS	White	None
2	70/male/OD	White	s/p CE; OAG
3	65/female/OD	Black	None
4	35/male/OS	Black	CME∗ treated with dorzolamide eye drops
5	57/male/OD	White	None
6	42/male/OS	White	None
7	49/female/OS	White	None

CME = nonangiographic cystoid macular edema due to retinitis pigmentosa; OAG = open-angle glaucoma, on 1 antiglaucoma medication; OD = right eye; OS = left eye; POH = past ocular history in study eye other than retinitis pigmentosa; s/p CE = pseudophakia.

∗ Nonangiographic cystoid macular edema in both eyes being treated with dorzolamide at baseline and continued during study follow-up.

As shown in Table 2, genetic testing results were available for all study participants except for participant #1 who had died from an accident 6 years after exiting from the study and was unavailable for genetic testing for RP when these tests became widely available. Participant #5 tested positive for 2 genetic variants of *EYS*, 1 pathogenic variant and 1 variant of uncertain significance. Participant #6 was homozygous for *USH2A* pathologic variant. Participant #7, who was suspected to have Usher’s syndrome based on hearing loss, tested positive for 2 genetic variants of *USH2A*, 1 pathologic variant and 1 variant of uncertain significance. All study participants received intravitreal injection of autologous CD34+ BMSCs in the study eye and completed the 6 months study follow-up.

**Table 2:** Genetic Testing Results of Study Participants

Participant	Known Pathogenic Variant	Variant of Uncertain Significance
1	n/a∗	n/a∗
2	None	-heterozygous for *KIAA1549* c.3584A>G, p.(Lys1195Arg); -heterozygous for *DHX38* c.1435A>G, p.(Met479Val)
3	-heterozygous for *PAX6* c.275G>A (p.Arg92Gln)	-heterozygous for following variants: *ALMS1* c.4798A>G (p.Ile1600Val), *CEP290*CEP290 c.4938A>G (silent), *CEP290*CEP290 c.5284C>T (p.Arg1762Cys), *HGSNAT* c.402C>G (p.Asn134Lys), *LRIT3* c.773C>T (p.Thr258Ile), *OCA2* Gain (exons1-23), *RCBTB1* c.725A>T (p.Tyr242Phe), *SLC45A2* c.1379C>T (p.Ala460Val), *TYRP1*c.823A>T (p.Thr275Ser), *VCAN* c.10064C>T (p.Pro3355Leu)
4	None	-heterozygous for following variants: *ADGRA3* c.20G>A (p.Arg7Gln), *GPR179* c.4277C>G (p.Ser1426Cys), *PEX5* c.667G>A (p.Gly223Ser), *SPATA7* c.1711A>G (p.Ser571Gly), *TRPM1* c.2162G>A (p.Arg721Gln), *USH1G*USH1G c.1340G>A (p.Arg447Gln)
5	-heterozygous *EYS* c.6714del (p.Ile2239Serfs∗17); -heterozygous *EYS* c.6958_6960del (p.Phe2320del)	-heterozygous *PDE6A* c.878C>T (p.Pro293Leu)
6	-homozygous *USH2A*USH2A c.10073G>A (p.Cys3358Tyr)	-heterozygous *ABCA4* c.6805C>T (p.Arg2269∗)
7	-heterozygous for *USH2A* c.3407G>A, p.(Ser1136Asn); -heterozygous for *ABCA4* c.5882G>A, p.(Gly1961Glu); -heterozygous for *MFSD8* c.103C>T, p.(Arg35∗)	- heterozygous for *USH2A* c.10586G>A, p.(Gly3529Asp);

∗ Participant died 6 years after exiting from study from an accident (pedestrian hit by a motor vehicle at night) and unavailable for genetic testing.

All study participants tolerated the bone marrow aspiration with a desired yield of bone marrow aspirate. The isolated CD34+ BMSCs (final product) from all study participants passed the release criteria. Thus, all study participants received intravitreal injection of autologous CD34+ BMSCs in the study eye. Table 3 summarizes the characteristics of the final cell product injected in the eye. The mean number of viable CD34+ BMSCs injected intravitreally was 3.26 ± 0.66 million (range, 1.6–7.05 million) based on the CliniMACs CD34+ cell isolation. The mean percentage of viable cells in the final released product based on trypan blue staining was 93.4% ± 1.7% (range, 87.0%–98.0%).

**Table 3:** CD34+ Cells Isolated from Bone Marrow and Injected Intravitreally as Study Treatment

Participant Number	Number of CD34+ Cells Injected Intravitreally (viable∗)	% Viability of Final Cell Product∗	% CD34+ Cells in Final Product by Flow Cytometry	Number of Viable CD34+ Cells Injected Intravitreally Based on Flow Cytometry	% CD3+ Cells in Final Product by Flow Cytometry
1	3.0 million	98.0%	68.96%	2.1 million	0.59%
2	3.1 million	94.8%	46.61%	1.4 million	1.56%
3	1.6 million	95.7%	60.69%	1.0 million	2.64%
4	2.98 million	87.0%	70.54%	2.1 million	1.61%
5	2.99 million	95.1%	83.99%	2.5 million	0.69%
6	7.05 million	87.0%	94.48%	6.7 million	0.45%
7	2.1 million	97.0%	92.17%	1.9 million	1.84%
Mean ± SEM	3.26 ± 0.66 million	93.4 ± 1.7 %	72.03 ± 6.56 %	2.1 ± 0.7 million	1.12 ± 0.30 %

SEM = standard error of the mean.

∗ Viability based on trypan blue staining of the isolated CD34+ cells before release.

Analysis of the remaining final cell product after it was released for intravitreal injection showed no concerns about sterility or purity. The 14-day sterility assay showed no growth in all cases. Postrelease analysis of the final cell product by flow cytometry revealed a mean of 72.03% ± 6.56% CD34+ cells (range, 94.48%–46.61%). This calculates to a mean number of viable CD34+ BMSCs of 2.1 ± 0.7 million (range, 1.0–6.7 million cells) injected per study eye based on flow cytometry identification of CD34+ cells in the final product. Postrelease flow cytometry analysis of the final product showed a mean of 1.12 ± 0.30 % CD3+ cells (range, 0.45%–2.64%), indicating a very low percentage of the isolated cells being T cells in the final cell products.

The intravitreal injection of autologous CD34+ BMSCs was well tolerated in all 7 participants during study follow-up with stable or improved BCVA at 6 months (Table 4). The only possible exception is study participant #7. In this participant, new cells were seen in the anterior chamber (AC) without flare on slit-lamp biomicroscopy conducted 18 hours after study cell injection. There was no eye pain or conjunctival injection but intraocular pressure was elevated to 30 mmHg. Best-corrected visual acuity was unchanged. This participant was reexamined 6 hours later, i.e., 24 hours after study injection, with complete resolution of the AC cells without intervention. A drop of timolol-dorzolamide was administered once in the study eye with normalization of the intraocular pressure 6 hours later. Timolol-dorzolamide was discontinued and intraocular pressure remained normal for the rest of study follow-up. Best-corrected visual acuity at 1 week and 4 weeks postinjection visit was decreased by 3 lines in both eyes in this participant with no subjective change in vision and no change in eye examination. Thereafter, BCVA in both eyes improved by 2 lines at 3 months and 6 months follow-up to a final BCVA which was 1 line below baseline in both eyes. No other changes were noted on eye examination and retinal imaging during study follow-up for all study participants.

**Table 4:** BCVA at Baseline and Study Follow-up in Study Eye

Participant Number/Study Eye	Baseline BCVA	BCVA at 1 Mo	BCVA at 3 Mos	BCVA at 6 Mos	BCVA∗ at Last Follow-up (Follow-up Duration)
1. OS	20/640	20/400	20/400−2	20/400−1	n/a
2. OD	20/50	20/40+1	20/40+1	20/50-1	LP (38 mos)
3. OD	20/25−1	20/20	20/25−1	20/20	20/60 (52 mos)
4. OS	20/32−2	20/32+1	20/32−1	20/32	20/200 (19 mos)
5. OD	20/100	20/125	20/200	20/80	n/a
6. OS	20/50−1	20/50−2	20/50−1	20/50−1	20/50 (13 mos)
7. OS	20/50	20/80−2	20/63	20/60−1	n/a

BCVA = best-corrected visual acuity; LP = light perception; n/a = not available; OD = right eye; OS = left eye.

∗ BCVA after study exit was Snellen visual acuity.

Figure S1 (available at www.ophthalmologyscience.org) shows baseline and 6 months follow-up fundus photographs of the study eye for all study participants. Figure S2 (available at www.ophthalmologyscience.org) includes baseline and 6 months follow-up macular OCT images of the study eye of all study participants. As shown, no change in fundus photography or macular OCT was noted in the study eye during the study follow-up in all study participants. Table 5 summarizes the central macular thickness on OCT showing a relatively stable measurement after 6 months when compared with baseline. Similarly, when the integrity of the photoreceptor layer is measured using a horizontal OCT scan centered at the fovea, the integrity of the photoreceptor layer measured slightly larger in 2 eyes and stable or slightly decreased in the remaining 5 eyes at 6 months follow-up when compared with baseline.

**Table 5:** Quantitation of CMT and Horizontal Width of Intact Photoreceptor Layer on Macular OCT

Participant	CMT, μm, Baseline	CMT, μm, 6 mos	Width of Intact IS/OS junction, mm, Baseline	Width of Intact IS/OS Junction, mm, 6 mos
#1	200	176	0.70	0.64
#2	254	254	1.15	1.04
#3	252	247	3.1	2.5
#4	310∗	322∗	1.4	1.2
#5	196	192	0.43	0.77
#6	287	277	0.66	0.55
#7	150	147	0.79	0.84

CMT = central macular thickness; mos, months; IS/OS junction = inner segment/outer segment junction of photoreceptor on OCT B-scan.

∗ Mild cystoid macular edema present on OCT.

### Perimetry

Perimetry was performed in all study participants at baseline and study follow-up (see Fig 1; see reference #16 for Goldmann perimetry for participant #1). Goldmann perimetry was conducted in 6 of the study participants. Participant #3 had Humphrey perimetry 24-2. Table 6 summarizes the perimetry results for the study eye and the contralateral eye (when test available) for baseline and study follow-up. For Goldmann perimetry, % area of sensitivity was measured for V4e and III4e (when available). For participant #3, mean deviation and pattern standard deviation are included for baseline and follow-up visits. The area of sensitivity on Goldmann perimetry at baseline was highly variable among the study eyes, ranging from 0.5% to 65% of the total area tested for the study eye. Nonetheless, the quantitation shows a trend toward an increase in area of sensitivity on Goldmann perimetry after study treatment in 5 of 7 participants (participant #1, 2, 4, 5, and 7) during study follow-up. For participants #5 and #7, a similar trend toward improvement was noted in the contralateral eye. In participant # 4, the area of sensitivity measured higher at 1 to 3 months, but lower at 6 months follow-up when compared with baseline (with similar changes noted in contralateral eyes). Quantitated perimetry parameters for participants #3 and #6 showed stable or possible slight worsening of sensitivity in the study eye during study follow-up when compared with baseline; similar trends were also observed in the contralateral eye (Table 6). No statistical analysis was performed to determine statistical significance of changes in perimetry given the small study sample size.

**Table 6:** Quantitation of Perimetry Results of Study Eye and Contralateral Eye at Baseline and Study Follow-up

Participant #--Laterality: Perimetry Test	% of Total Test Area (Baseline)	% of Total Test Area (1 to 3 Mos)	% of Total Test Area (6 Mos)
#1 – OS: V4e	65%	70%	75%
OS: III4e	35%	27%	56%
Contralateral eye (OD)	n/a	n/a	n/a
#2 – OD: V4e	0.5%	0.65%	1.9%
Contralateral eye (OS): V4e	0.44%	0.46%	0.37%
#3 – OD: HVF-24-2∗	MD −13.9; PSD 11.18	MD −15.33; PSD 11.04	MD −14.83; PSD 11.23
Contralateral eye (OS): HVF 24-2∗	MD −7.09 PSD 5.24	MD −7.40 PSD 5.48	MD −7.3 PSD 5.59
#4 – OS: V4e	34.1%	44.9%	20.0%
Contralateral eye (OD): V4e	47.0%	55.0%	28.7%
#5 – OD: V4e	4.70%	6.80%	6.68%
Contralateral eye (OS)	4.00%	4.41%	6.81%
#6 – OS: V4e	1.28%	0.98%	0.95%
Contralateral eye (OD)	1.88%	0.89%	0.84%
#7 – OS: V4e	45.50%	50.0%	47.56%
OS: III4e	17.63%	17.24%	22.83%
Contralateral eye (OD): V4e	39.8%	58.2%	51.0%
OD: III4e	20.4%	15.7%	18.9%

HVF = Humphrey Visual Field; MD = mean deviation; OD = right eye; OS = left eye; PSD = pattern standard deviation.

∗ Based on Humphrey perimetry (24-2). All other participants had Goldmann perimetry with % area of sensitivity based on total test area.

### Vision Questionnaire

Because 2 of the first 3 study participants (participants #1 and #2) voluntarily reported possible subjective improvement in peripheral vision after cell injection that resulted in them performing activities that they were unable to do before study treatment, we modified the study protocol to include the National Eye Institute vision questionnaire to be conducted at baseline and at study exit for all subsequent study participants. The vision questionnaire was performed for the last 4 study participants at baseline and at 6 months follow-up (Table 7). The score improved in all 4 participants by a mean of 5.5 (range, 2–12); 50% (2 of 4 participants) had improvement of >4 points, which is considered significant.

**Table 7:** NEI Visual Function Questionnaire Results at Baseline and Study Exit

Participant	Visual Function Questionnaire Score at Baseline	Visual Function Questionnaire Score at 6 Mos, Study Exit
Participant #4	78	80
Participant #5	76	88
Participant #6	79	82
Participant #7	73	88

NEI = National Eye Institute.

### Long-term Follow-up

After study exit at month 6, long-term follow-up information (i.e., clinical information >6 months after study exit) was available in 4 participants who returned to the study center for follow-up eye examination as part of standard of care for their eye condition. The follow-up ranged from 13 to 52 months. As shown in Table 4, the most recent BCVA (Snellen) in the study eye was worse after study exit in 3 of the 4 study participants due to progression of RP. Participant #2 developed rapid loss of vision in the study eye with subluxation of the posterior chamber lens implant within 2 years after study exit. The contralateral eye of this participant had a posterior chamber intraocular lens implant in place but with loose zonules. The subluxed lens implant was removed surgically in the study eye but BCVA remained poor postoperatively. The decreased vision was attributed to progression of RP.

## Discussion

This phase I, open-label study showed that intravitreal injection of autologous CD34+ BMSCs is feasible and relatively well tolerated in eyes with vision loss from RP. Each study participant tolerated both the bone marrow aspiration and intravitreal injection of autologous CD34+ BMCS in the study eye. A high yield of viable CD34+ cells could be isolated under good manufacturing practice conditions from the bone marrow aspirate of each study participant for intravitreal injection despite the bone marrow aspiration being performed under local anesthesia and as an outpatient. The cell injection was well tolerated during the 6 months study follow-up for all study participants. The current study findings are consistent with our previous report showing safety and feasibility of intravitreal injection of autologous CD34+ BMSC as preliminary findings among study participants with vision loss from various different retinopathies.^16^ A larger phase I/II randomized, prospective, sham-controlled double masked study is currently ongoing to further evaluate the safety, feasibility, and potential efficacy of this cell therapy in eyes with vision loss from central retinal vein occlusion.^9^

Our current study found 1 participant with a transient mild elevation in intraocular pressure with new cells in the AC when examined 18 hours after study cell injection. These cells in the AC were not associated with signs of intraocular inflammation, such as flare, eye pain, or eye injection. The cells in the AC fully resolved without intervention when reexamined 6 hours later. They likely represented transient anterior migration of the injected CD34+ BMSCs in this patient who may have had some zonular dehiscence from old eye trauma. This same study participant also was noted with a 3-line decrease in BCVA in both eyes at 1 week and 1-month follow-up which improved at 3 months and 6-month follow-up. The variable BCVA in this participant was not associated with subjective worsening of vision and likely related to suboptimal fixation in this patient. Macular OCT imaging showed disruption of the photoreceptor layer close to the fovea in both eyes in this study participant at baseline and at study follow-up (Fig S2, available at www.ophthalmologyscience.org).

Our current study was not designed to evaluate for efficacy because it is a small, open-label study. Nonetheless, it is encouraging to note that a majority of the study participants noted some subjective improvement in visual function based on the questionnaire which was conducted at baseline and study exit. Similarly, quantitative analysis of area of sensitivity of 6 study participants who had Goldmann perimetry showed stable or increased area of sensitivity in all study eyes during study follow-up, except for 1 study eye (participant #4) showing initial increase with subsequent decrease at study exit compared with baseline which was also observed in the contralateral eye. Participant #3 who had Humphrey perimetry had stable or slightly worsened mean deviation during study follow-up which was also observed in the contralateral eye. When improvement in perimetry was noted in the study eye, similar changes were also observed in the contralateral eye in 3 of the 6 participants where contralateral eye information is available. Although this may reflect a placebo or learning effect, contralateral eye effect of the study treatment cannot be ruled out because some of the injected CD34+ cells potentially may migrate into the systemic circulation and home into other damaged tissue.^10^

This is noteworthy because 6 of the 7 study participants had visual field constriction up to 10°, a sign of advanced RP. Electroretinography testing showed a flat signal in all study eyes at baseline which remained unchanged after cell injection in all eyes. OCT imaging of the macula also showed a relatively stable macular thickness at study exit compared with baseline. Similarly, macular photoreceptor layer integrity appeared relatively stable at study exit when compared with baseline.

Other functional tests such as mobility testing and full-field stimulus threshold testing may be more sensitive in detecting function recovery in patients with advanced RP than perimetry or ERG. This was noted in the gene therapy clinical trials for *RPE65*-associated RP.^6^ Mobility testing and full-field stimulus threshold testing were not available at the study center for the current clinical trial but will be considered in any future larger randomized, sham-controlled clinical trial evaluating efficacy of this cell therapy for RP.

Functional preservation of the retina has been observed in preclinical models of RP after intravitreal injection of human CD34+ BMSCs. In Royal College of Surgeons rats with hereditary retinal degeneration, immunosuppressed with cyclosporin to avoid rejection of human cells, the ERG signal was transiently preserved after a single intravitreal injection of human CD34+ BMSCs.^14^ No preservation of retina morphology was demonstrated on histology at 4 weeks after intravitreal injection of human CD34+ BMSC injection. The transient nature of the functional preservation of the retina after intravitreal injection of CD34+ cells in this rodent model may be due to eventual rejection of human cells because cyclosporin does not provide full immunosuppression. It also may reflect the true transient effect of CD34+ cells injected intravitreal in eyes with a progressive degenerative condition. In rd1 mice with rapidly progressive advanced hereditary retinal degeneration, immunosuppressed systemically with rafamycin and tacrolimus, rapid retinal homing of the human CD34+ cells after intravitreal injection was noted with dramatic molecular changes in the retina despite lack of functional rescue.^12^ The molecular changes were in retinal genes that regulate apoptosis and photoreceptor transduction and maintenance.

Among our study participants with extended follow-up after exiting the study, progressive vision loss was noted in both eyes in 3 of the 4 participants. Similarly, in our previous report, an eye with geographic atrophy from age-related macular degeneration was noted with progression of geographic atrophy during the study follow-up despite improvement in visual acuity during the 6 months study follow-up.^16^ Whether repeat intravitreal injection of CD34+ BMSCs will result in an enhanced or more durable therapeutic effect is unknown at the current time. It is a question that should be explored in future clinical trials. Currently, repeat intravitreal injection of allogeneic fetal progenitor cells is being explored in a phase II clinical trial for RP.^9^

There are multiple ongoing clinical trials exploring various types of cellular therapies to treat retinal degeneration, but the use of intravitreal injection of CD34+ BMSCs offers several unique advantages.^8^^,^^10^ First, we can use autologous cells that will not be rejected or cause intraocular inflammation. In addition, CD34+ cells are minimally manipulated cells that are not actively dividing. This results in minimal risk of abnormal cellular proliferation in the eye after injection, a safety concern that has been observed using cultured cells. Furthermore, CD34+ cells home into damaged tissue and promote tissue repair via paracrine mechanism. Thus, we do not have to perform vitrectomy surgery to inject the cells subretinal to have therapeutic effects. By concentrating CD34+ cells and injecting these effector cells directly into the eye, we aim to maximize the repair potential of these cells on the degeneration retina using a minimally invasive procedure. Multiple preclinical and clinical studies have been conducted showing that enrichment of CD34+ cells in the final product, as done in our study, results in superior tissue repair effect when compared with using unpurified mononuclear cells from bone marrow or peripheral blood.^10^^,^^11^

Our study has several limitations. First, the final cell product injected intravitreal was enriched with CD34+ stem cells and not pure CD34+ cells. Based on flow cytometry, the mean concentration of CD34+ cells in the final product was 72%. Thus, it can be argued that any therapeutic effect resulting from using the final cell product may be due to cells other than CD34+ cells in the final product. Although this possibility cannot be ruled out, multiple large cardiology clinical trials have been performed using unpurified monocular cell fraction or CD34+ enriched cell product (similar to our study) from bone marrow or peripheral blood for tissue regeneration. These studies show that the therapeutic effects of the cell therapy are more consistently observed using the CD34+ cell enriched product.^10^^,^^11^ The procedure used in our study to isolate CD34+ cells is a standard that has been used to isolate CD34+ cells for bone marrow transplantation and for other clinical trials.^10^^,^^11^^,^^16^^,^^17^

Additional study limitations include a small sample size and long enrollment period, both resulting from limited funding support. Although all enrolled participants were diagnosed with RP based on clinical examination, flat ERG, and history of progressive loss of night and peripheral vision, it should be noted that none of the study participants had known family history of RP and genetic testing did not confirm the diagnosis of RP in all study participants. This is not surprising because not all genetic mutations associated with RP have been identified and mode of inheritance of RP is variable.^1^^,^^7^ Nonetheless, our study provided important additional information regarding feasibility and safety of this novel cell therapy in eyes with RP which potentially can be used to design future larger clinical trials.

An additional study limitation is that the study protocol was modified slightly during the course of the study. Enrollment BCVA and perimetry criteria were modified to increase study enrollment because we noted a high rate of screen failures during the early phase of the study. In addition, we limited perimetry to Goldmann perimetry during the course of the study for consistency. Perimetry was limited to the study eye and not consistently performed in the contralateral eye for comparison in the early phase of the study. Lastly, the vision questionnaire was conducted only for the last 4 study participants and only after the first few participants volunteered information regarding possible changes in activities of daily living after study enrollment. Nonetheless, the vision questionnaire showed a consistent trend for increase in the functional score among the study participants after study enrollment, a potential efficacy signal for this small open-label study.

In summary, our phase I clinical trial showed safety and feasibility of intravitreal injection of autologous CD34+ BMSCs in eyes with vision loss from RP. Although the study was small and not designed to evaluate for efficacy based on the open-label design, some possible efficacy signals were observed on perimetry and vision questionnaire that warrant further investigation. A larger randomized prospective study incorporating other potentially more sensitive functional testing, such as full-field stimulus threshold and mobility testing, is warranted to fully evaluate safety and efficacy of this novel cell therapy for RP.
