# Supplementary material for: Phase I Study of Intravitreal Injection of Autologous CD34+ Stem Cells from Bone Marrow in Eyes with Vision Loss from Retinitis Pigmentosa
Source: Ophthalmol Sci. 2024 Jul 31;5(1):100589. doi: 10.1016/j.xops.2024.100589 (PMC11426125; doi:10.1016/j.xops.2024.100589)

Supplement Figure 1a: Fundus photography of the study eye at baseline and at 6 months follow-up showing no change after study cell injection. Participant #1 at baseline (A) and at 6 months (B). Participant #2 at baseline (C) and at 6 months (D).

A

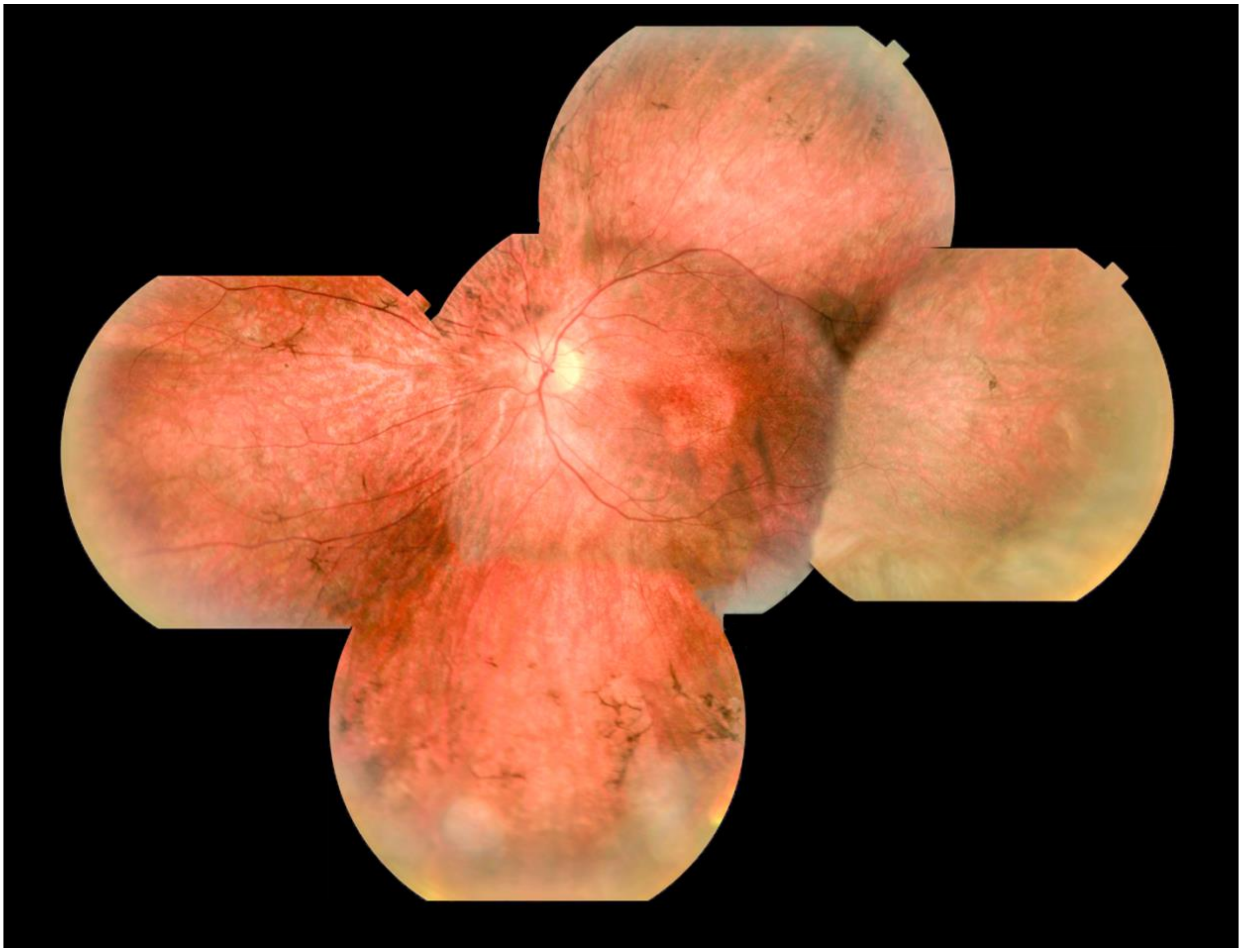

B

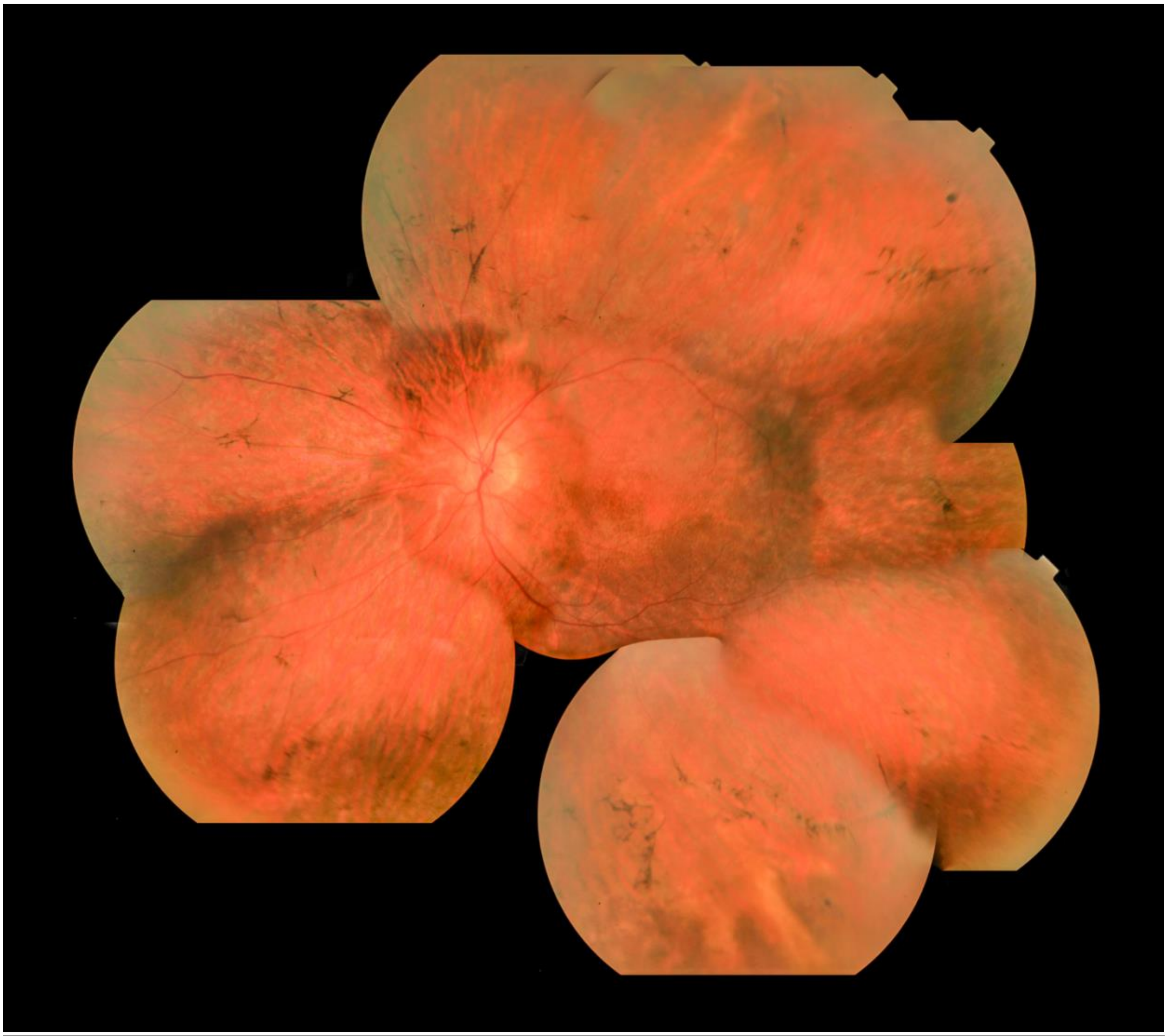

C

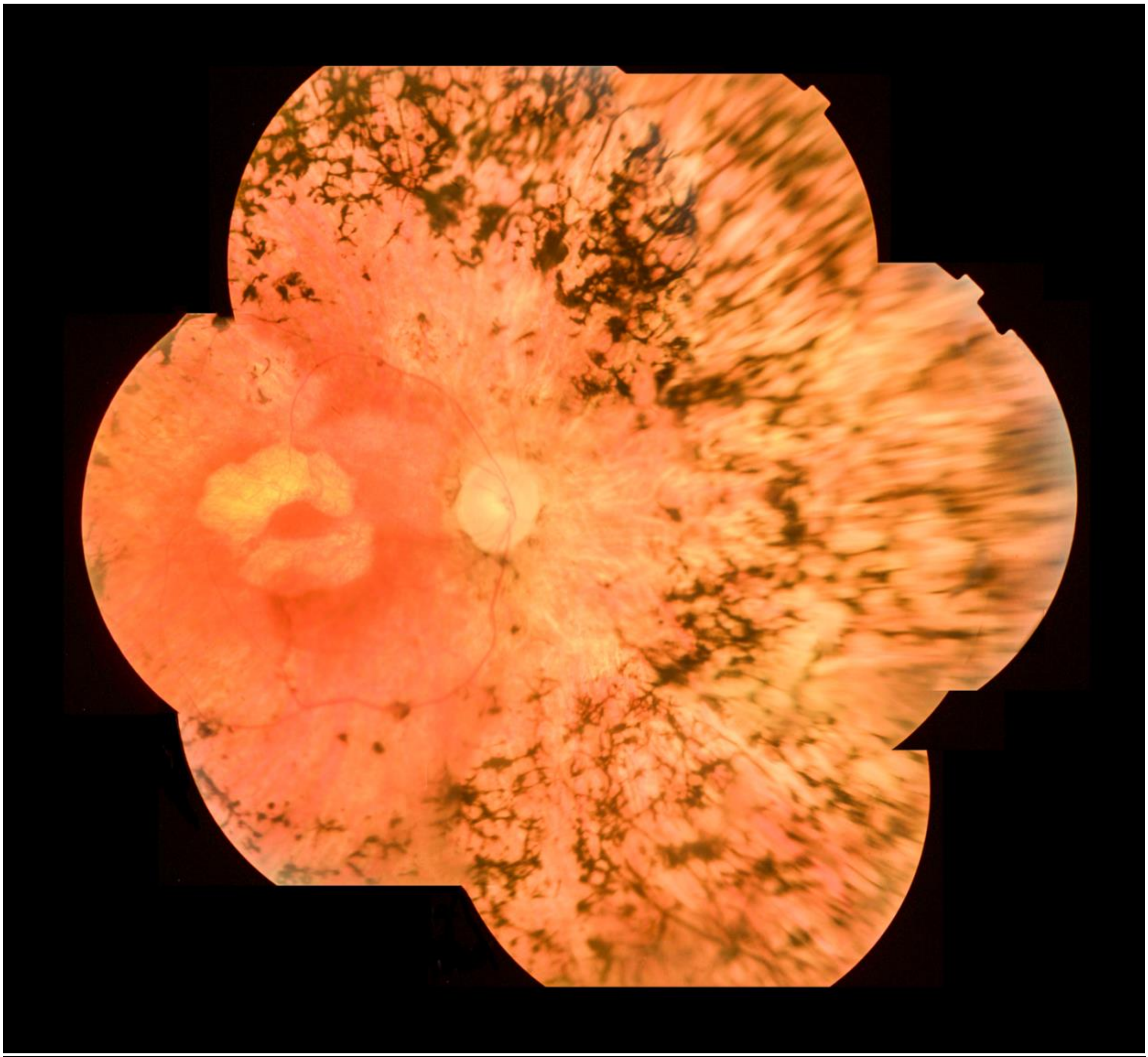

D

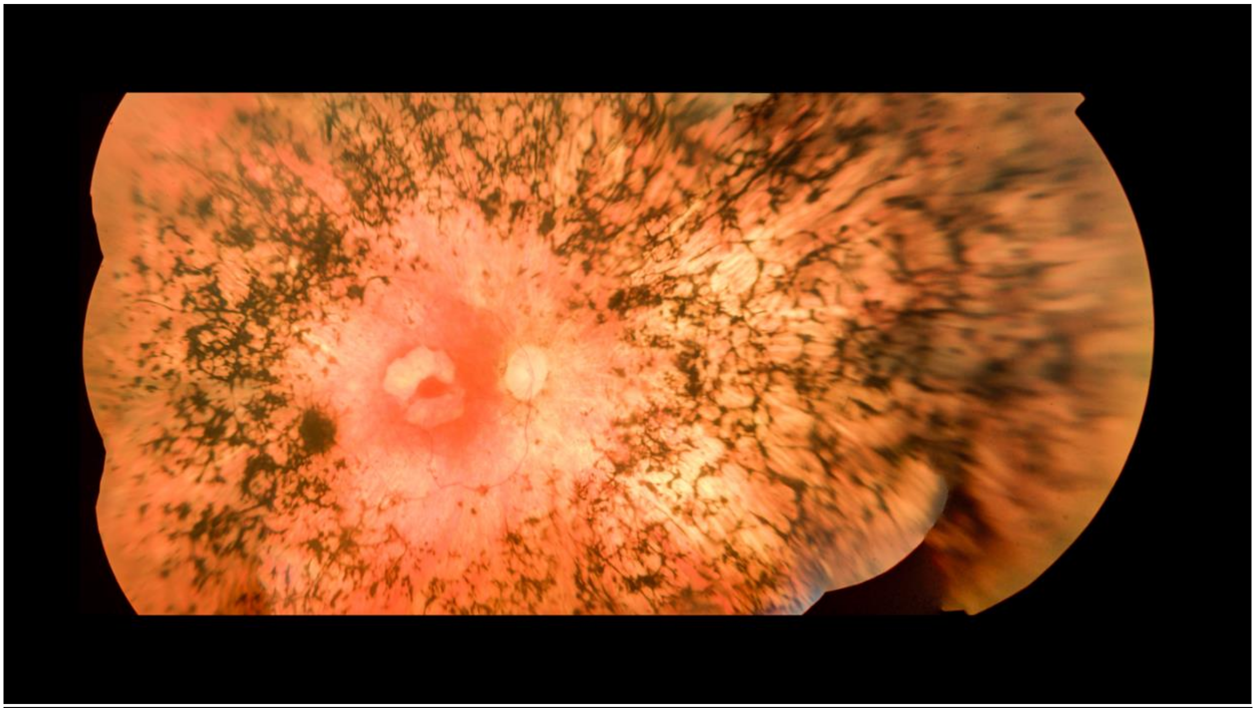

Supplement: Supplement Figure 1a [file mmc1.pdf]
