# Supplementary material for: Phase I Study of Intravitreal Injection of Autologous CD34+ Stem Cells from Bone Marrow in Eyes with Vision Loss from Retinitis Pigmentosa
Source: Ophthalmol Sci. 2024 Jul 31;5(1):100589. doi: 10.1016/j.xops.2024.100589 (PMC11426125; doi:10.1016/j.xops.2024.100589)

Supplement Figure 1c: Fundus photography of the study eye at baseline and at 6 months follow-up showing no change after study cell injection. Participant #5 at baseline (I) and at 6 months (J). Participant #6 at baseline (K) and at 6 months (L).

I

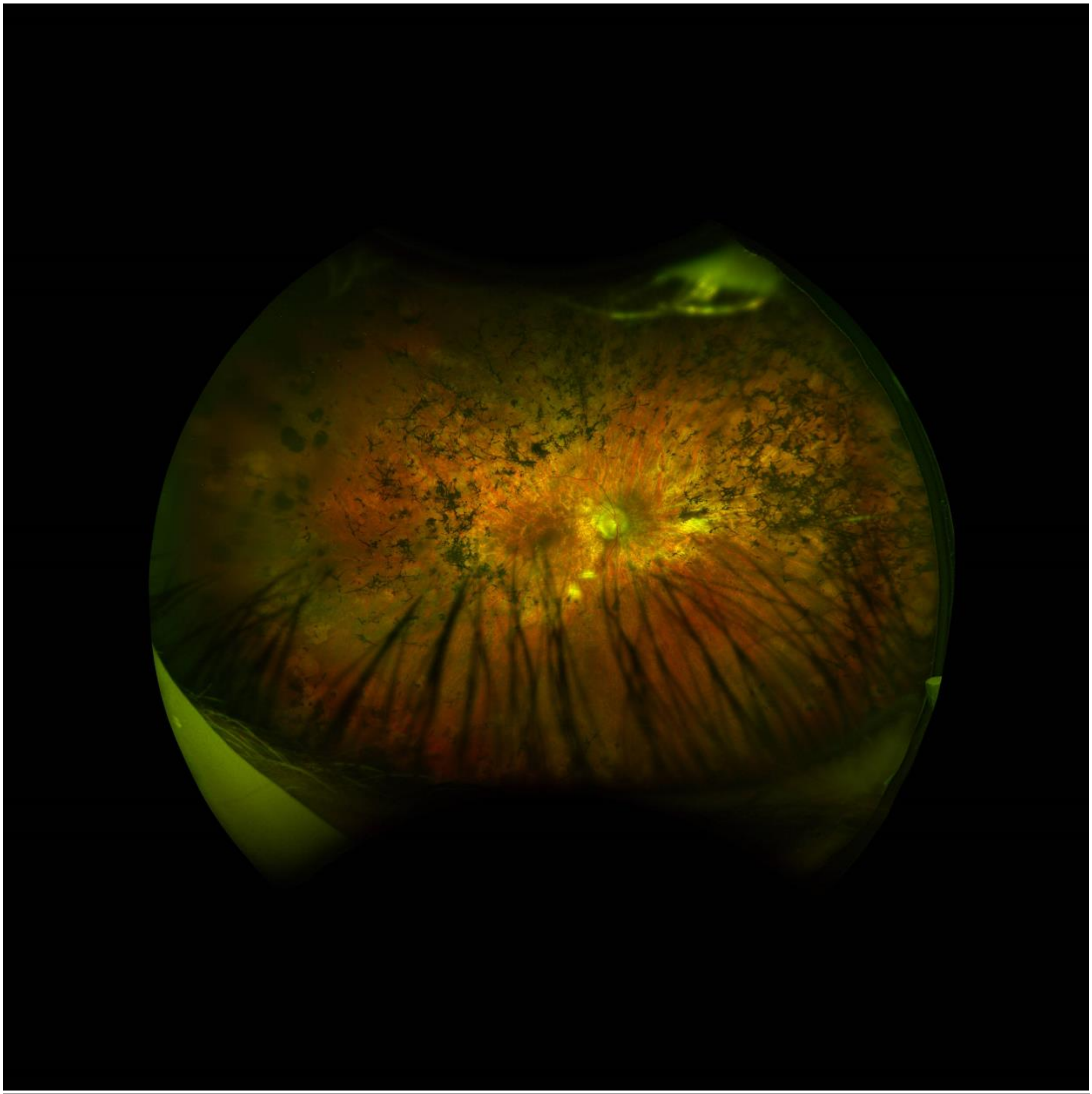

J

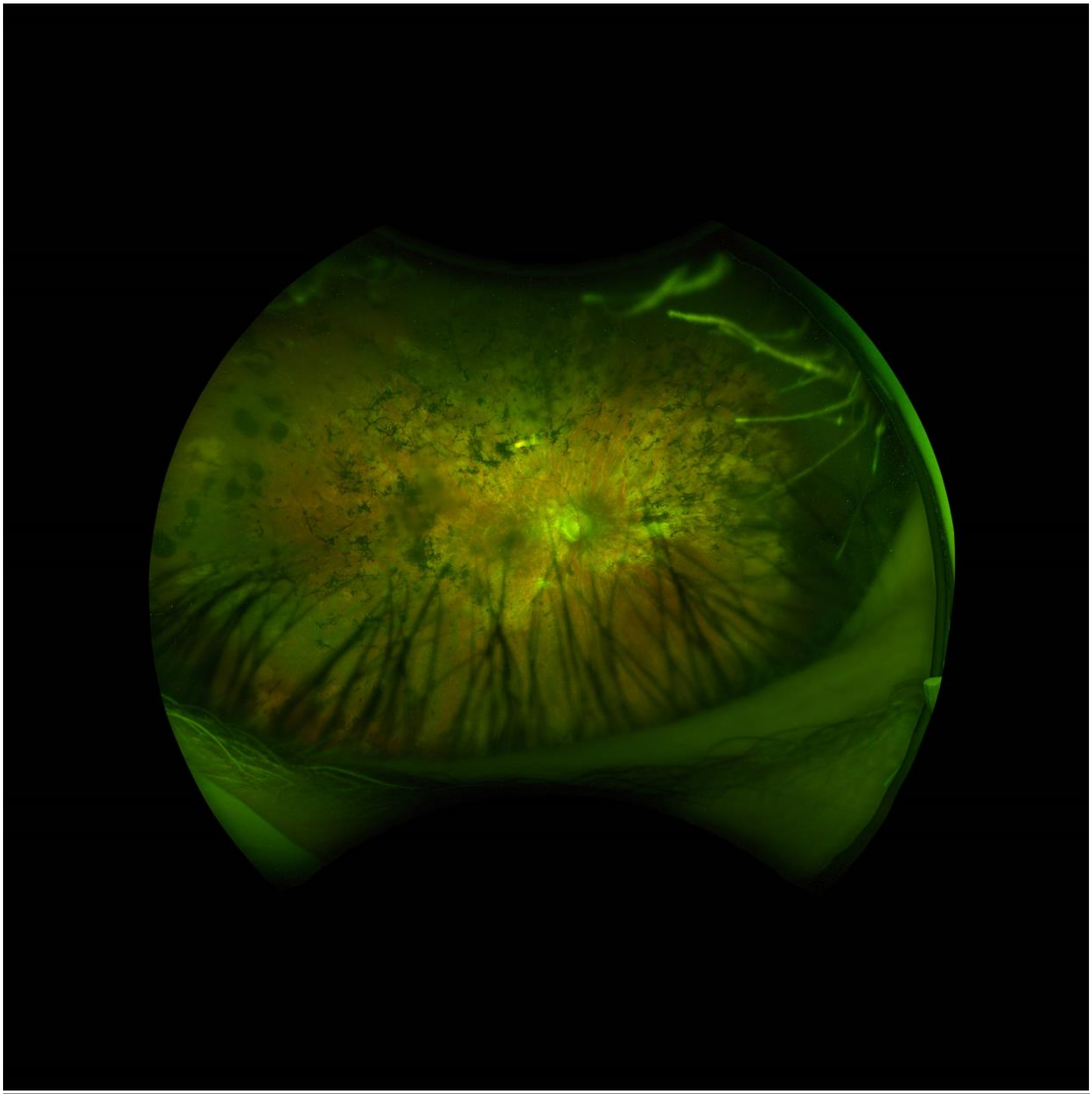

K

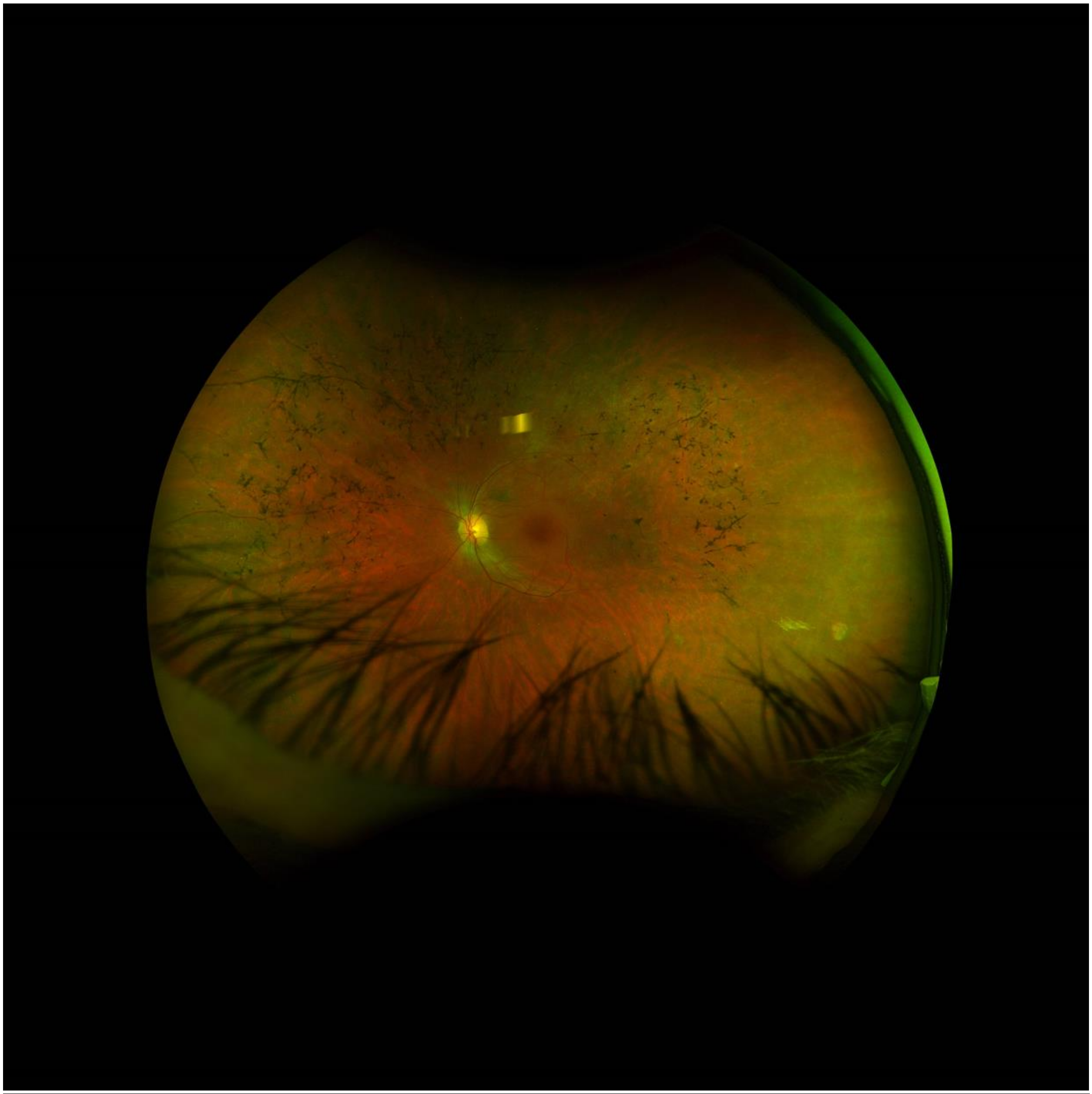

L

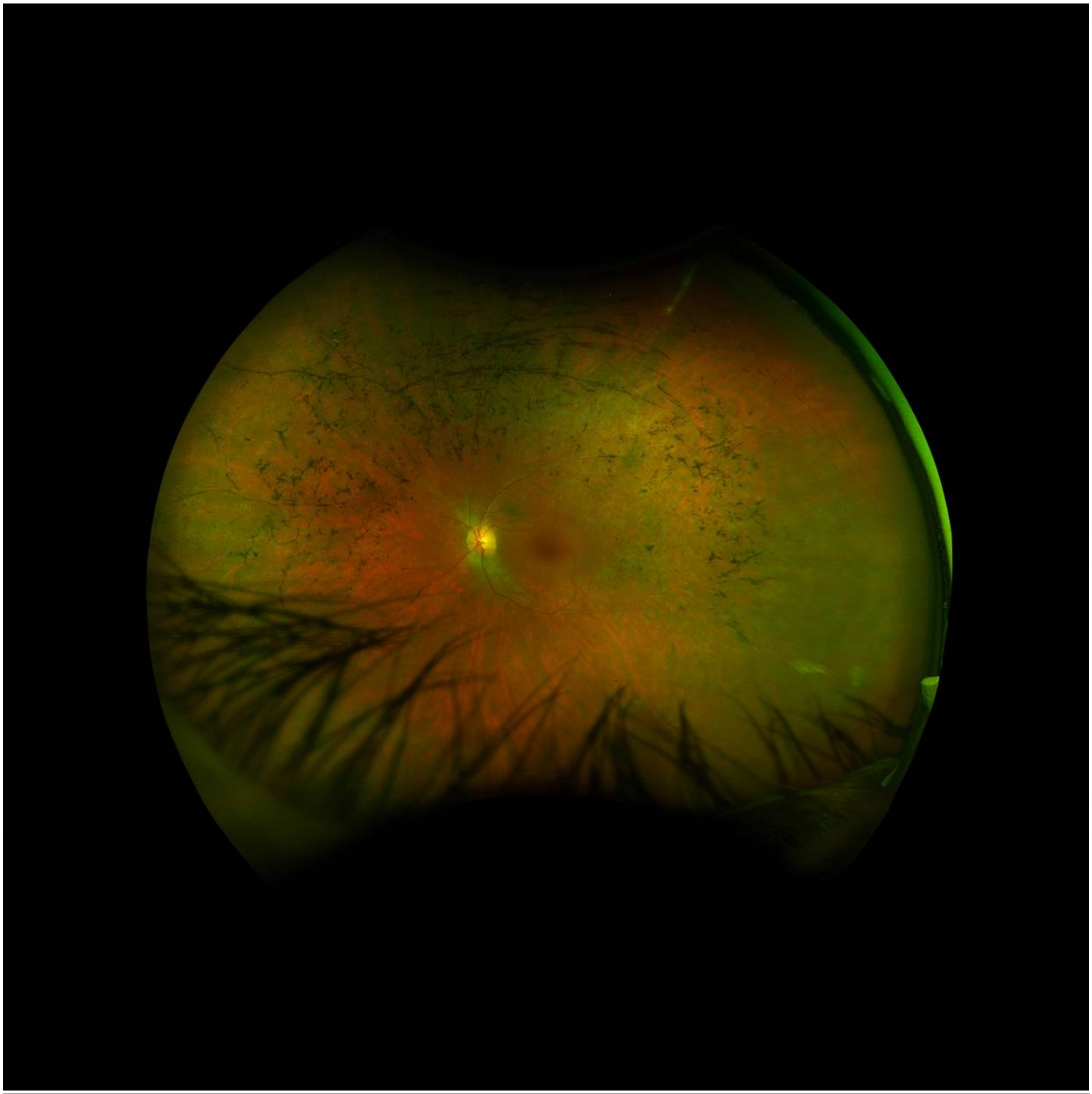

Supplement: Supplement Figure 1c [file mmc3.pdf]
