# Supplementary material for: Phase I Study of Intravitreal Injection of Autologous CD34+ Stem Cells from Bone Marrow in Eyes with Vision Loss from Retinitis Pigmentosa
Source: Ophthalmol Sci. 2024 Jul 31;5(1):100589. doi: 10.1016/j.xops.2024.100589 (PMC11426125; doi:10.1016/j.xops.2024.100589)

<u>Supplement Figure 1d</u>: Fundus photography of the study eye at baseline and at 6 months follow-up showing no change after study cell injection. Participant #7 at baseline (M) and at 6 months (N).

<u>M</u>

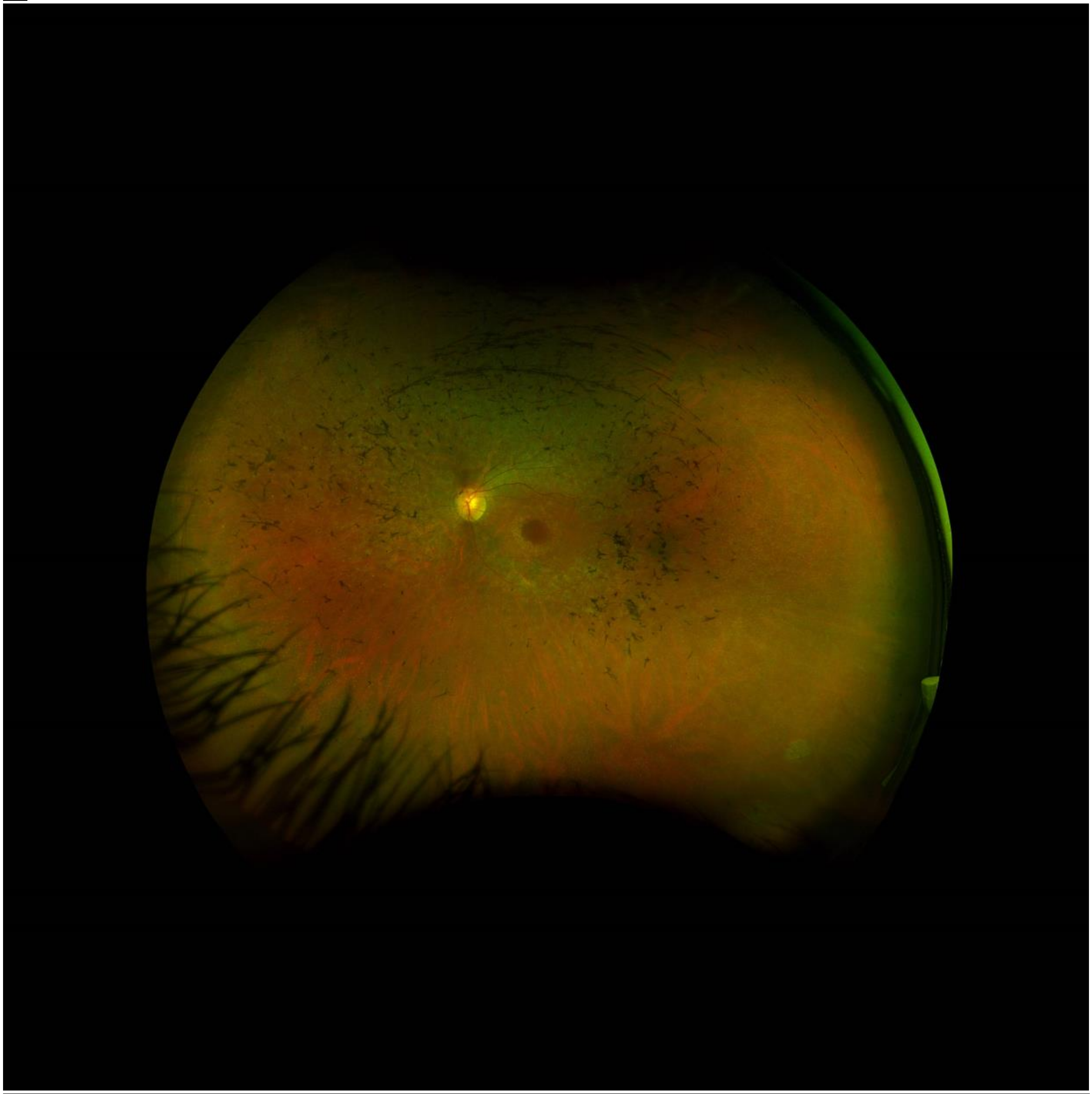

N

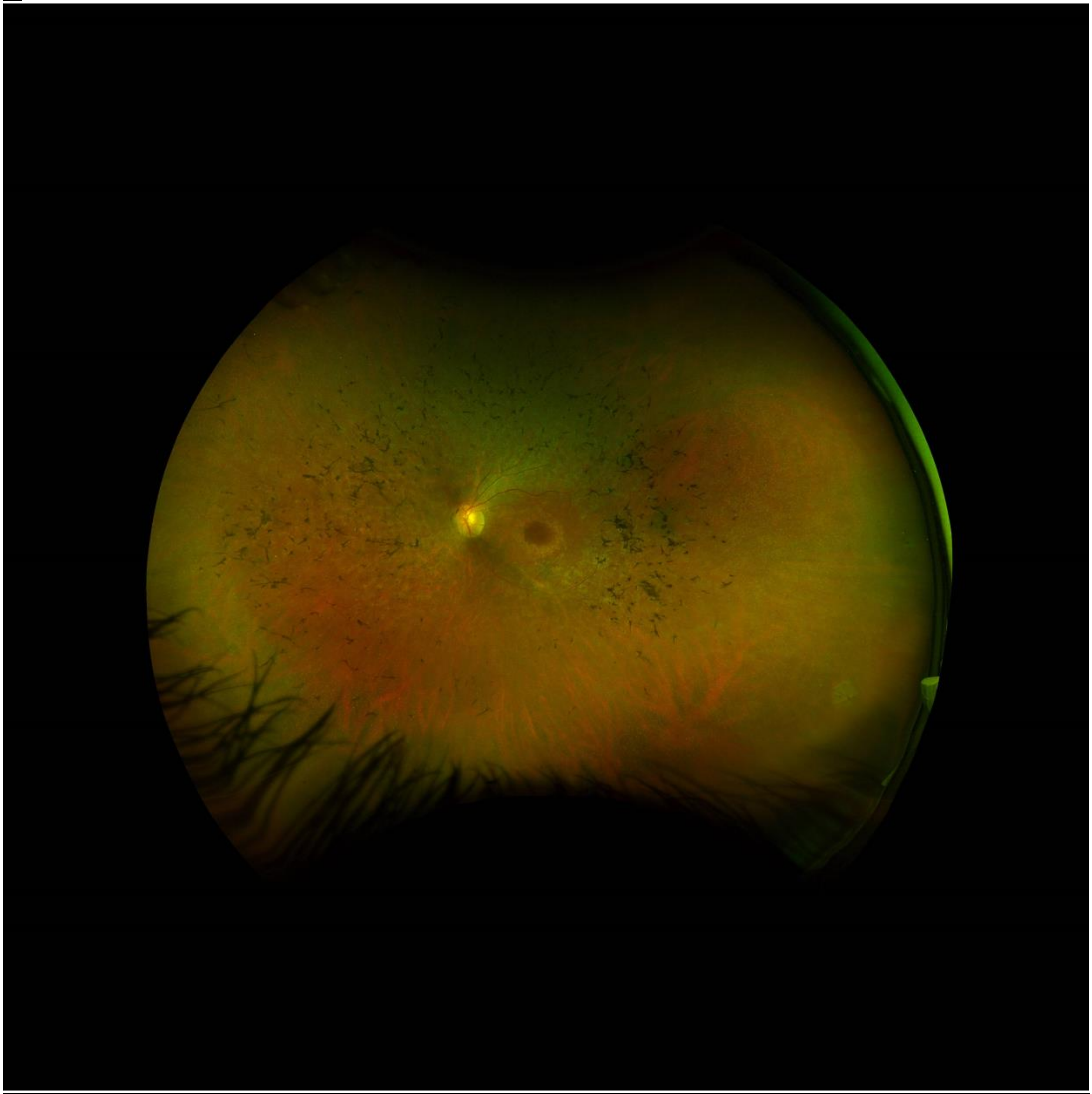

Supplement: Supplement Figure 1d [file mmc4.pdf]
