# Supplementary material for: Phase I Study of Intravitreal Injection of Autologous CD34+ Stem Cells from Bone Marrow in Eyes with Vision Loss from Retinitis Pigmentosa
Source: Ophthalmol Sci. 2024 Jul 31;5(1):100589. doi: 10.1016/j.xops.2024.100589 (PMC11426125; doi:10.1016/j.xops.2024.100589)

Supplement Figure 2: Horizontal B-scan OCT image of the macula of the study eye at baseline and 6 months study follow-up. Participant #1 at baseline (A) and at 6 months (B). Participant #2 at baseline (C) and at 6 months (D); Participant #3 at baseline (E) and at 6 months (F). Participant #4 at baseline (G) and at 6 months (H). Participant #5 at baseline (I) and at 6 months (J). Participant #6 at baseline (K) and at 6 months (L). Participant #7 at baseline (M) and at 6 months (N).

A

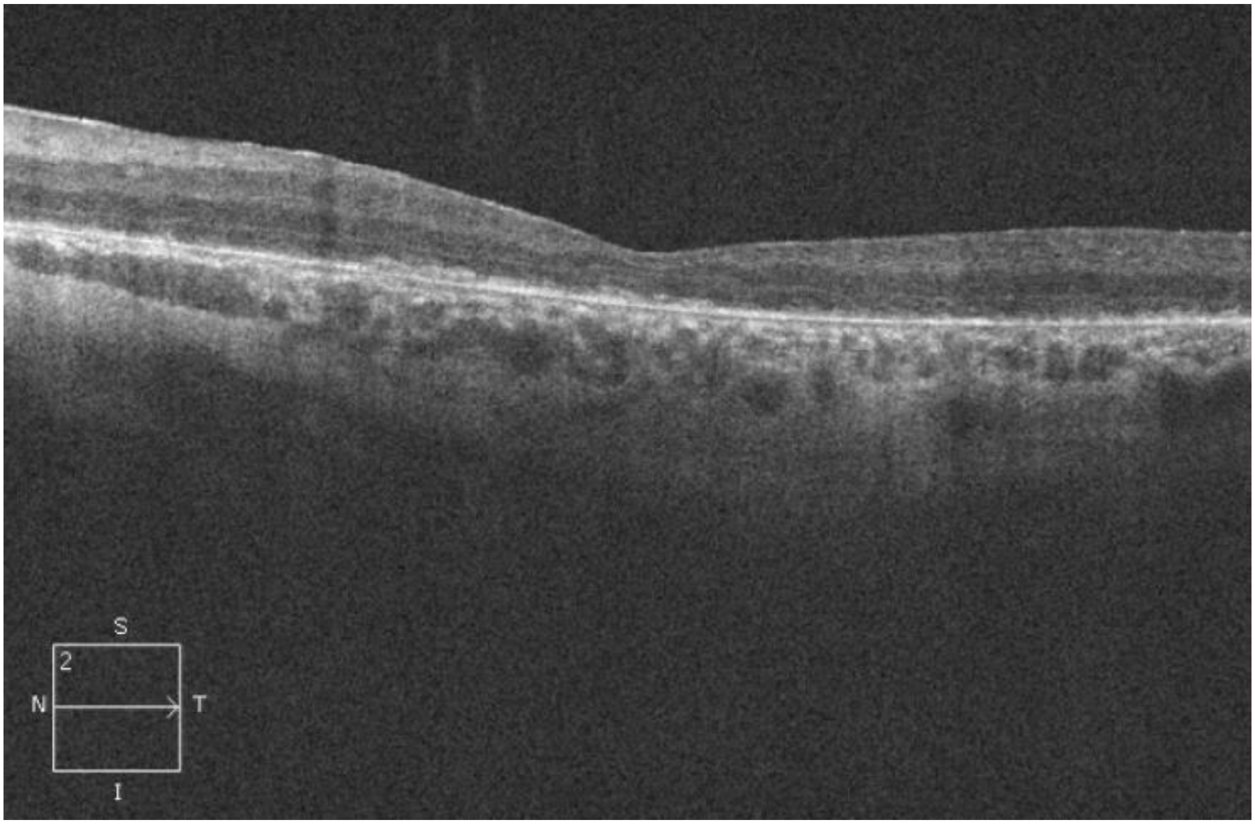

B

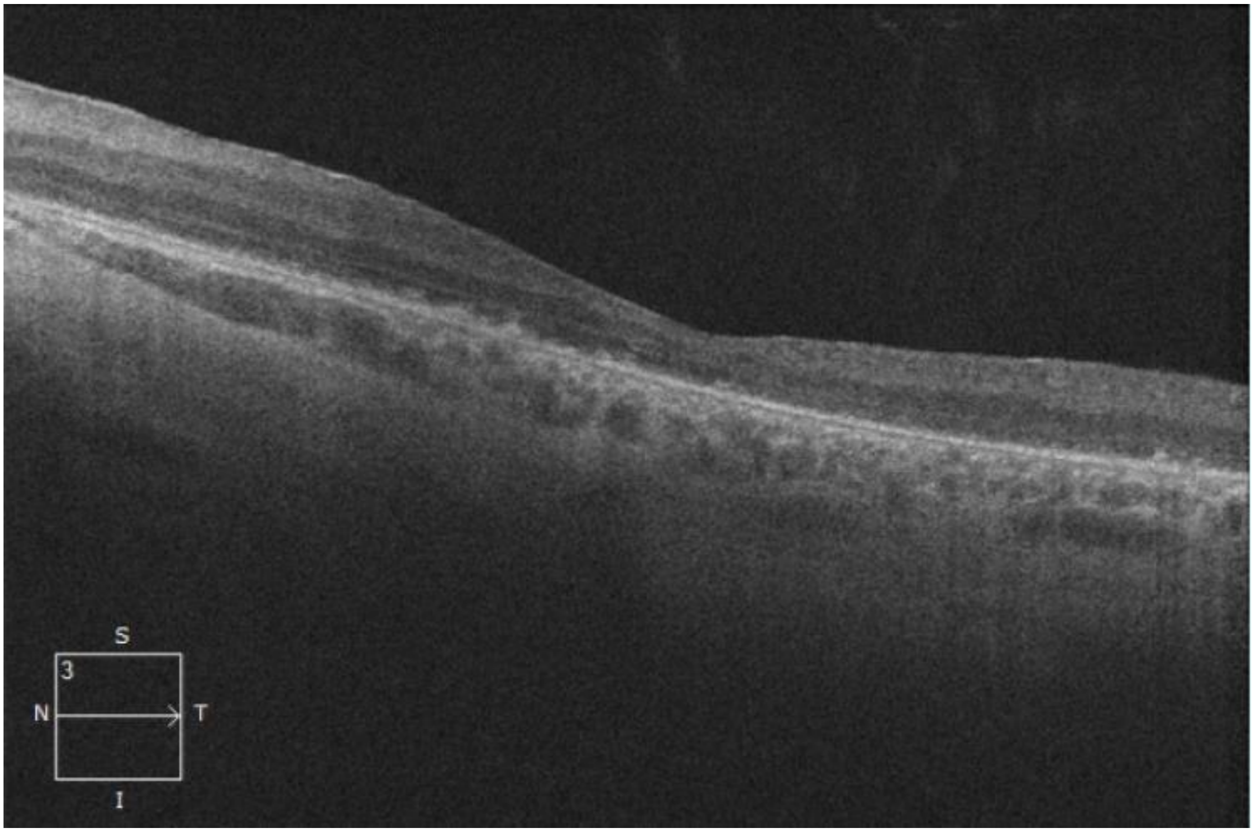

C

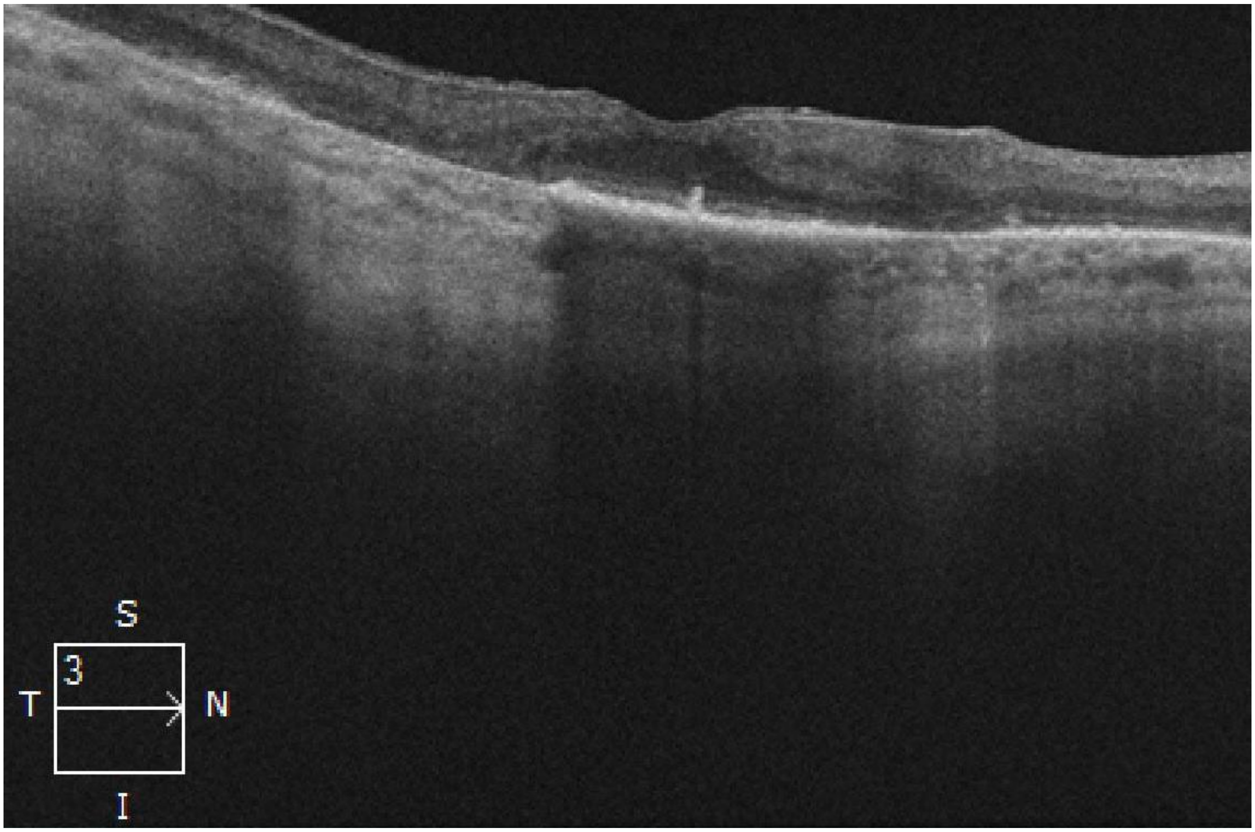

D

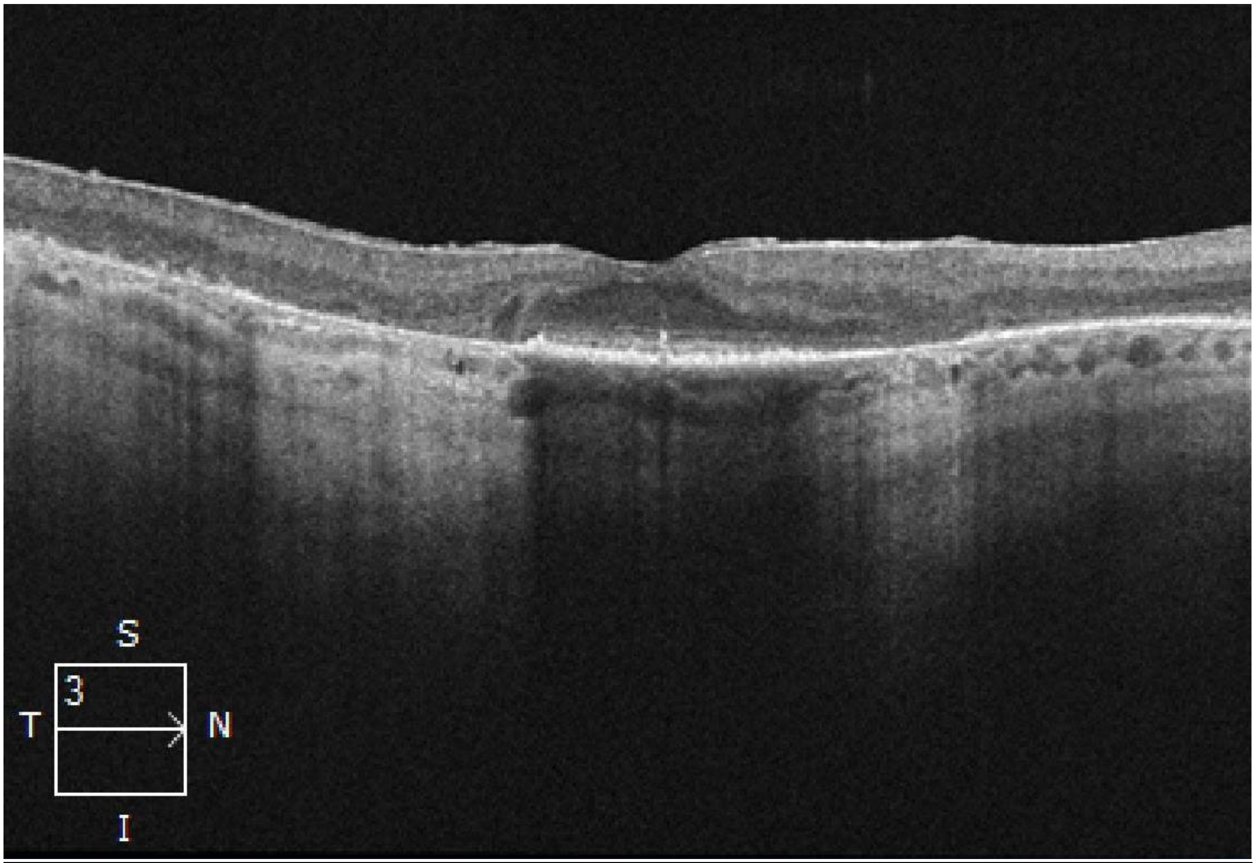

E

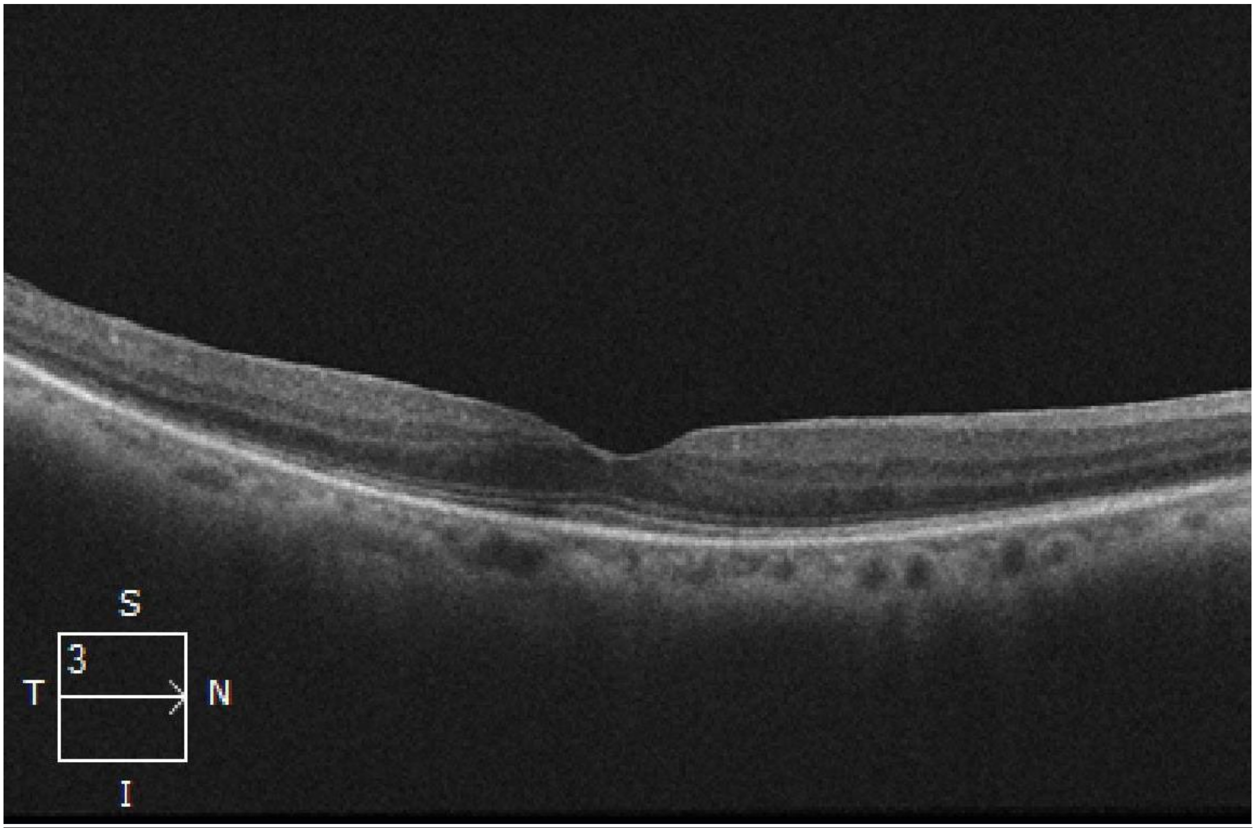

F

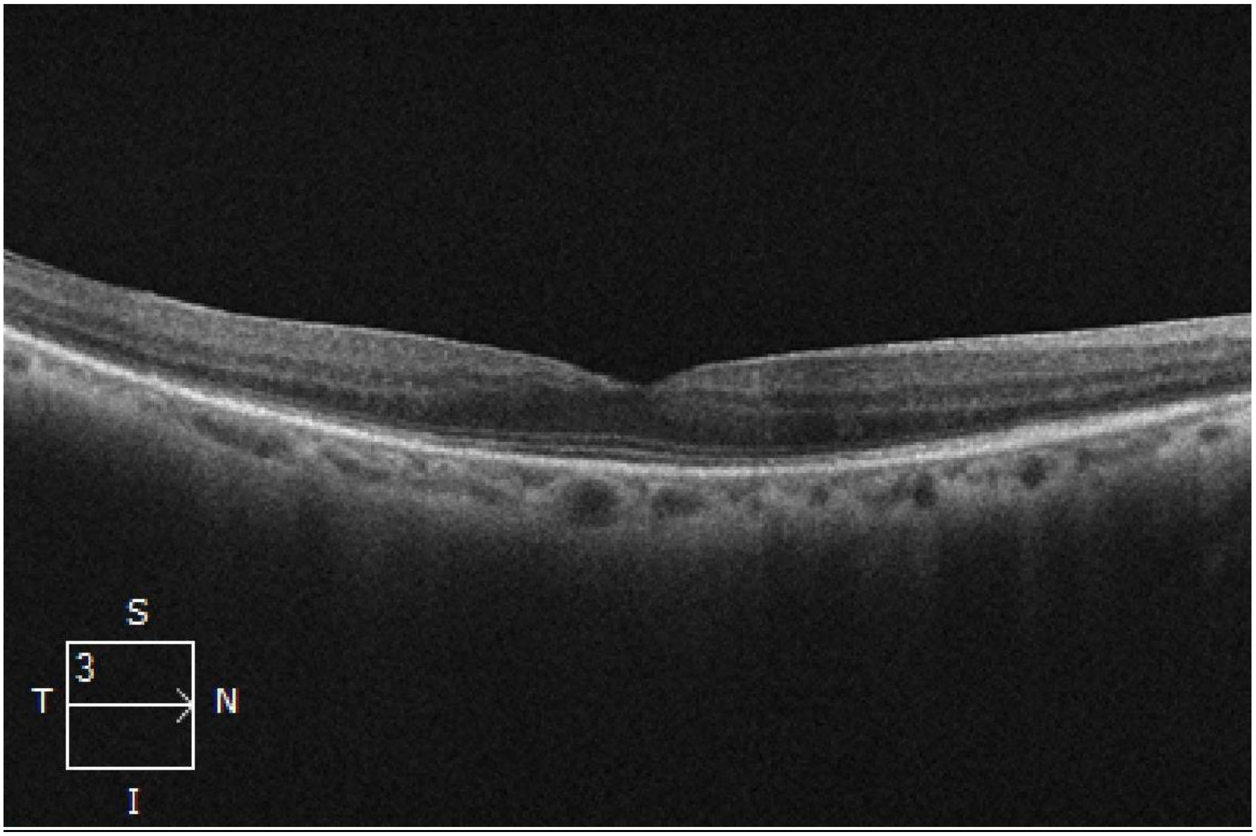

G

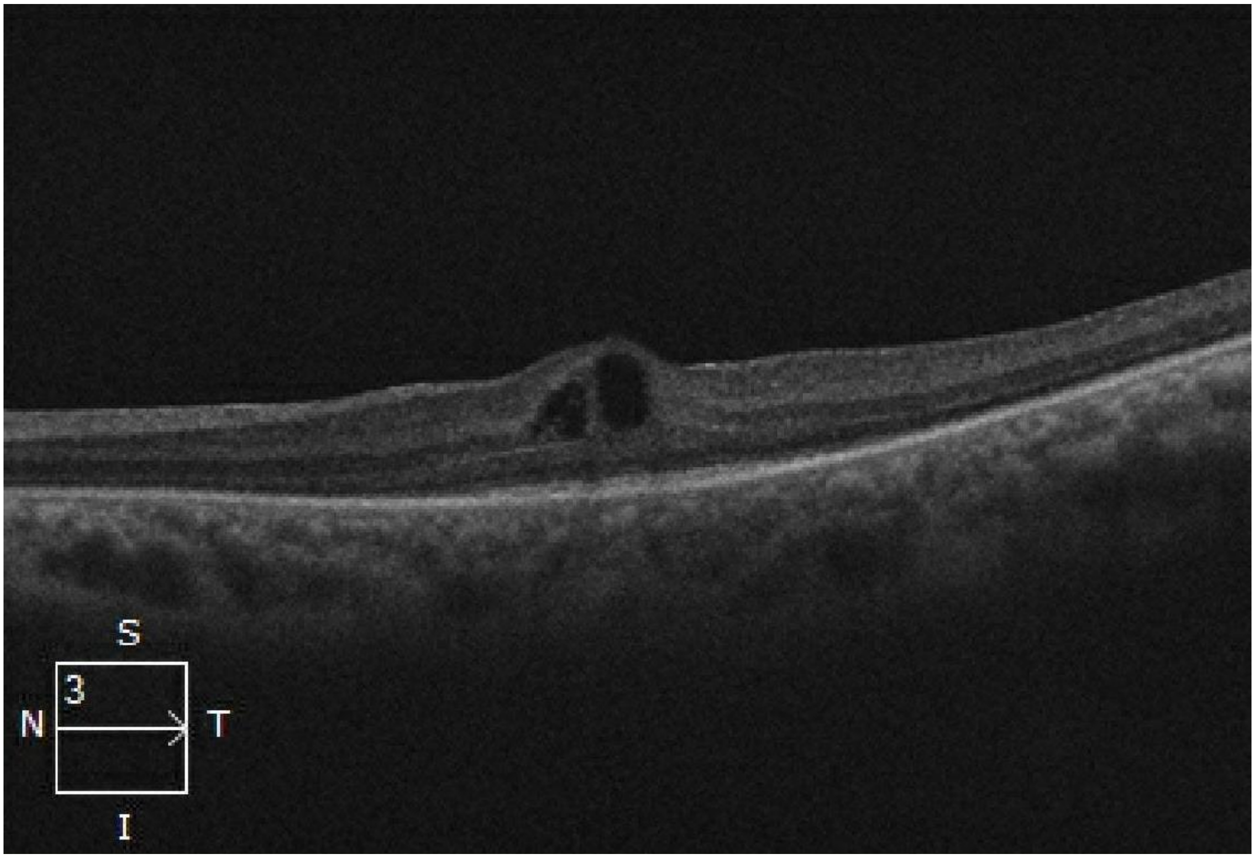

H

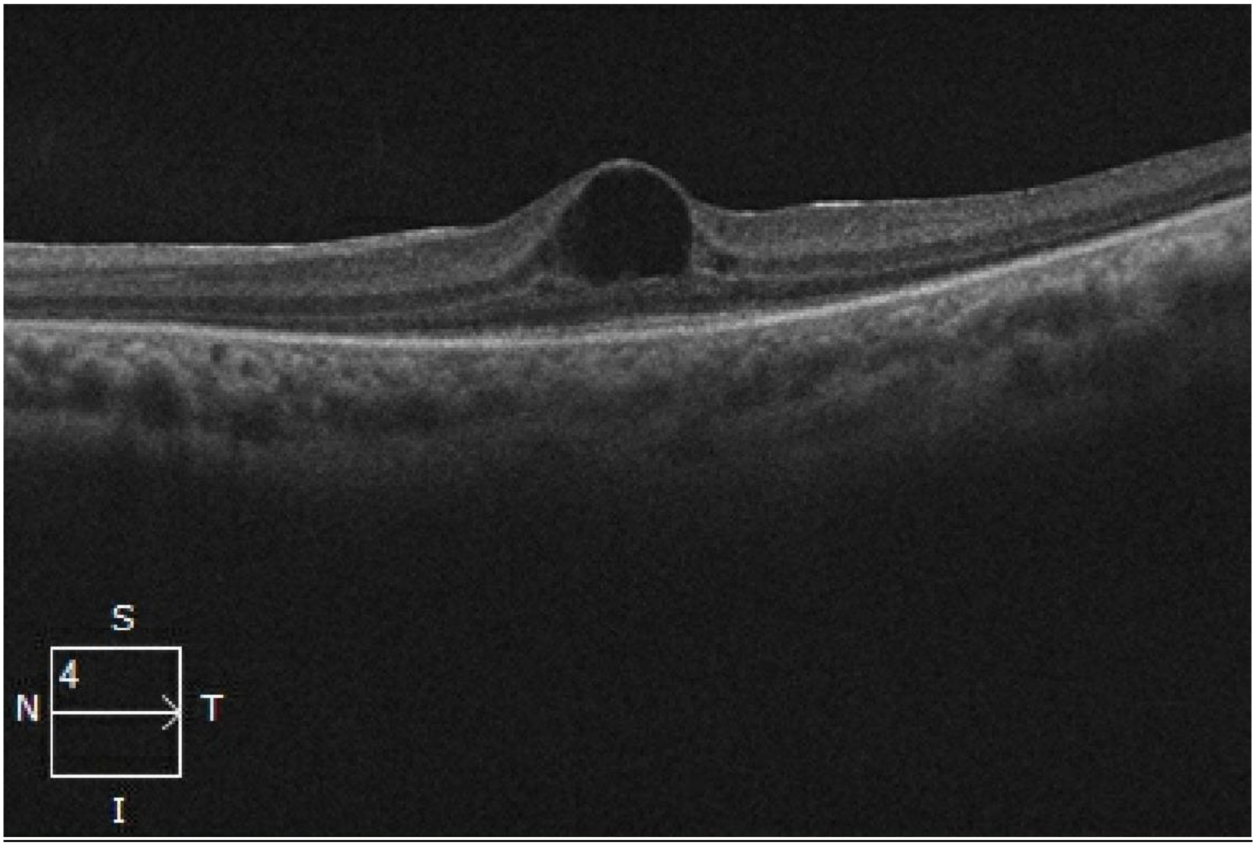

I

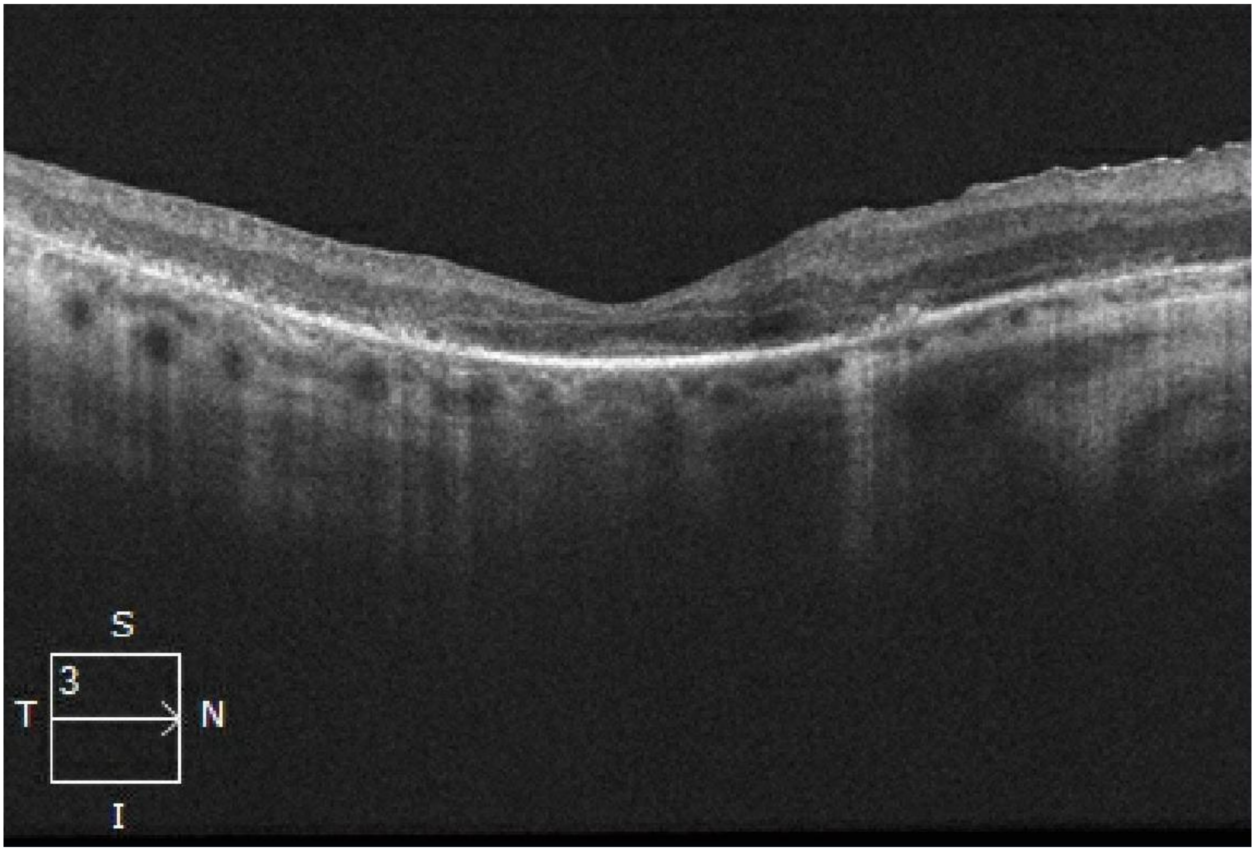

J

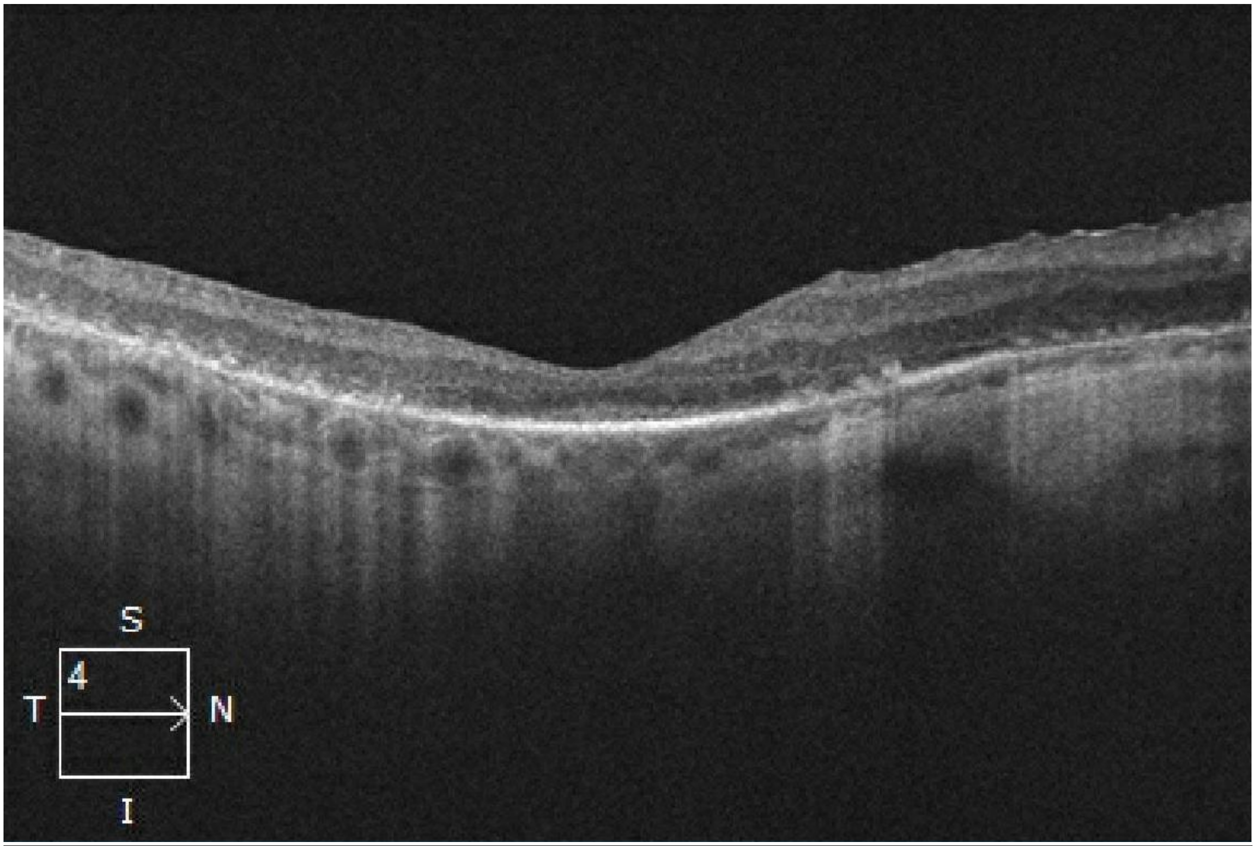

K

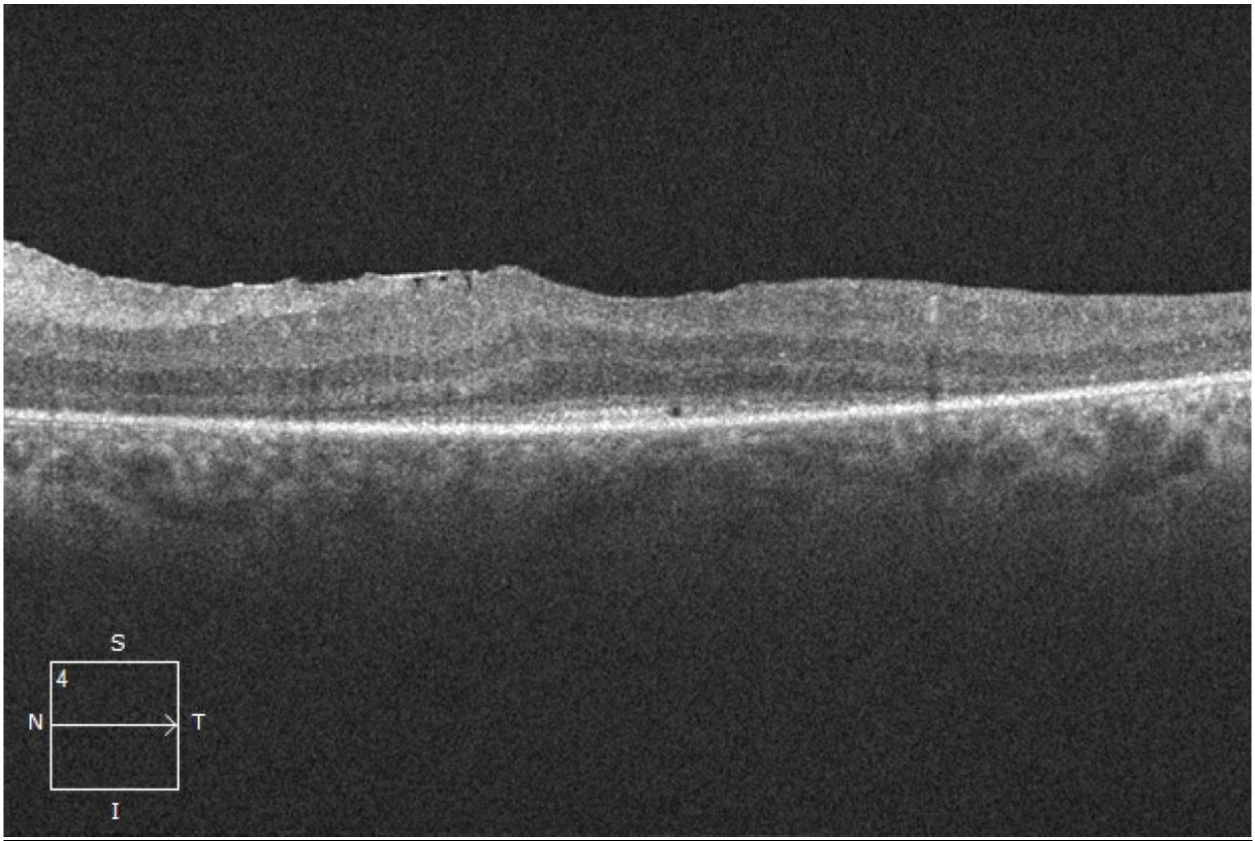

L

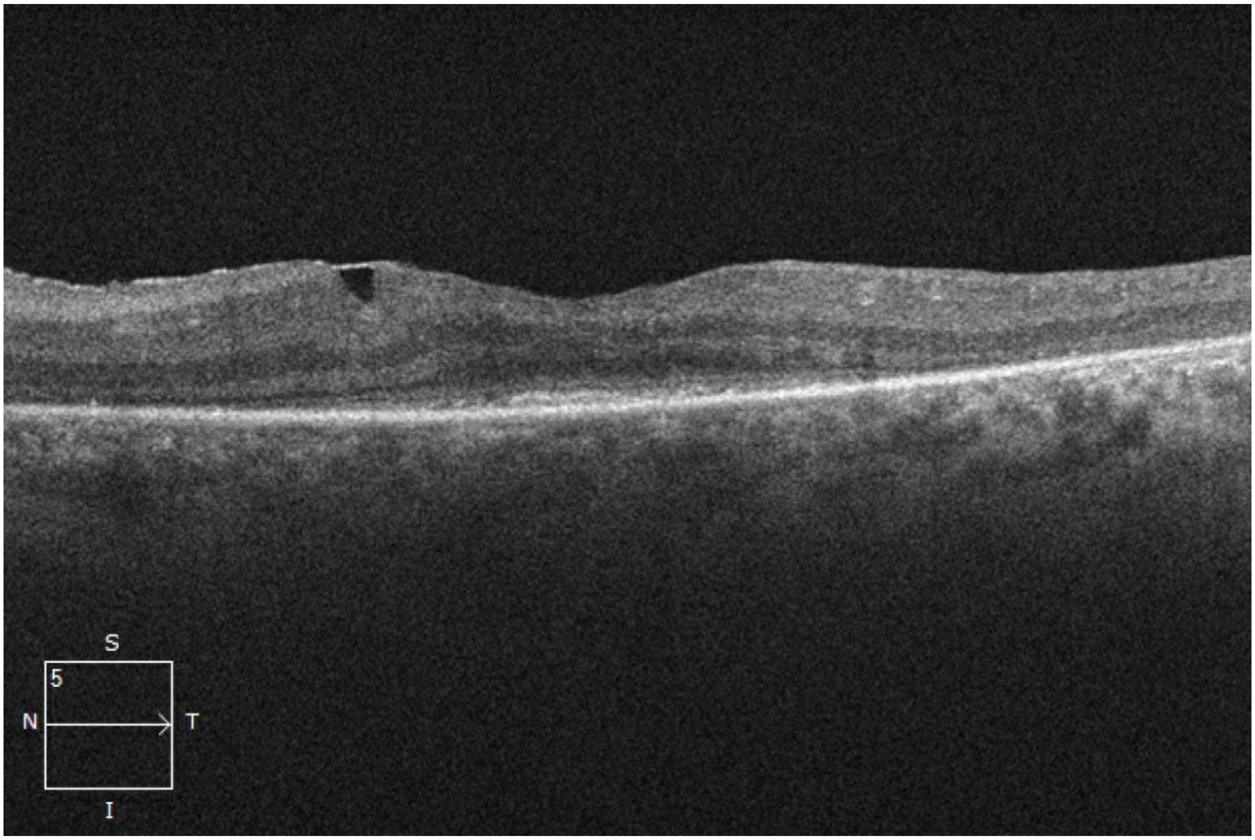

M

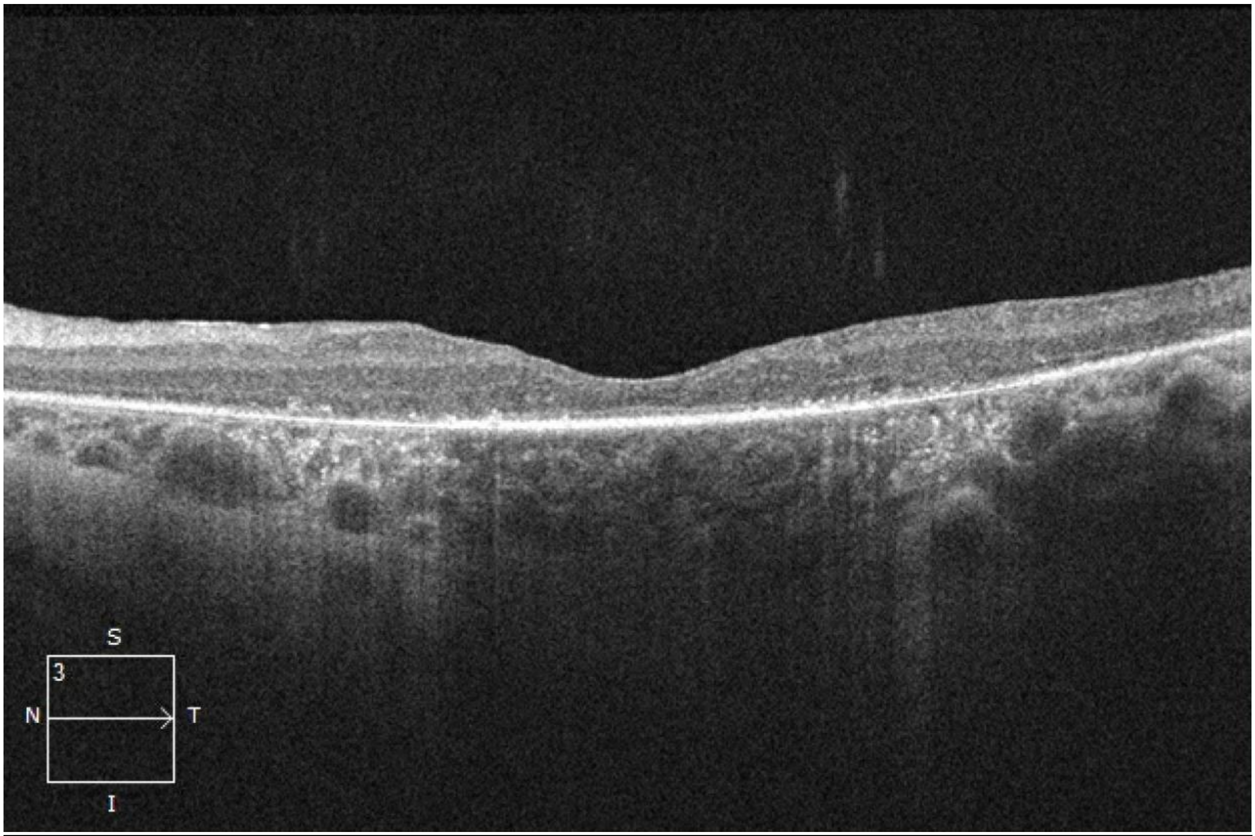

N

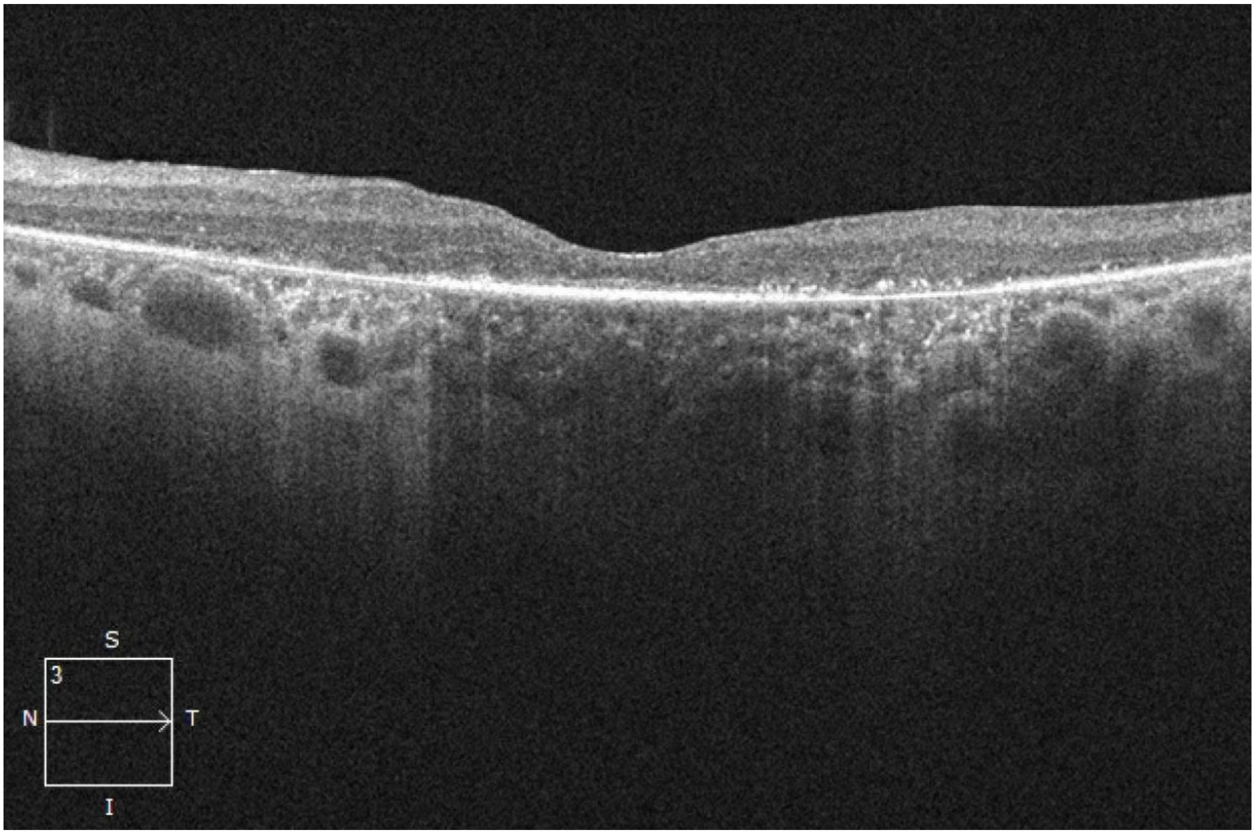

Supplement: Supplement Figure 2 [file mmc5.pdf]
